# Informing Investment to Reduce Inequalities: A Modelling Approach

**DOI:** 10.1371/journal.pone.0159256

**Published:** 2016-08-03

**Authors:** Andrew McAuley, Cheryl Denny, Martin Taulbut, Rory Mitchell, Colin Fischbacher, Barbara Graham, Ian Grant, Paul O’Hagan, David McAllister, Gerry McCartney

**Affiliations:** 1 Public Health Science Directorate, NHS Health Scotland, Glasgow, United Kingdom; 2 Information Services Division, NHS National Services Scotland, Edinburgh, United Kingdom; 3 Public Health, NHS Fife, Kirkcaldy, United Kingdom; 4 Centre for Population Health Sciences, University of Edinburgh, Edinburgh, United Kingdom; New York City Department of Health and Mental Hygiene, UNITED STATES

## Abstract

**Background:**

Reducing health inequalities is an important policy objective but there is limited quantitative information about the impact of specific interventions.

**Objectives:**

To provide estimates of the impact of a range of interventions on health and health inequalities.

**Materials and Methods:**

Literature reviews were conducted to identify the best evidence linking interventions to mortality and hospital admissions. We examined interventions across the determinants of health: a ‘living wage’; changes to benefits, taxation and employment; active travel; tobacco taxation; smoking cessation, alcohol brief interventions, and weight management services. A model was developed to estimate mortality and years of life lost (YLL) in intervention and comparison populations over a 20-year time period following interventions delivered only in the first year. We estimated changes in inequalities using the relative index of inequality (RII).

**Results:**

Introduction of a ‘living wage’ generated the largest beneficial health impact, with modest reductions in health inequalities. Benefits increases had modest positive impacts on health and health inequalities. Income tax increases had negative impacts on population health but reduced inequalities, while council tax increases worsened both health and health inequalities. Active travel increases had minimally positive effects on population health but widened health inequalities. Increases in employment reduced inequalities only when targeted to the most deprived groups. Tobacco taxation had modestly positive impacts on health but little impact on health inequalities. Alcohol brief interventions had modestly positive impacts on health and health inequalities only when strongly socially targeted, while smoking cessation and weight-reduction programmes had minimal impacts on health and health inequalities even when socially targeted.

**Conclusions:**

Interventions have markedly different effects on mortality, hospitalisations and inequalities. The most effective (and likely cost-effective) interventions for reducing inequalities were regulatory and tax options. Interventions focused on individual agency were much less likely to impact on inequalities, even when targeted at the most deprived communities.

## Introduction

Health inequalities can be defined as, ‘the systematic differences in the health of people occupying unequal positions in society’ and exist across a wide range of social factors including gender, income, deprivation and geography [1]. Galea and colleagues estimated that social factors in the United States explained a large proportion of premature mortality and stressed the need for an approach to public health policy and practice that addresses the wider determinants of health [2]. Recent research by Eikemo et al aimed to quantify the potential for reducing inequalities by addressing a number of key determinants, including both ‘upstream’ (i.e. lack of social participation, low income, and economic inactivity) and ‘downstream’ (i.e. smoking, overweight, and physical inactivity) risk factors [3], although we recognise the complex reality of causal pathways [4]. They concluded that there is a substantial potential for reducing inequalities through implementation of equity-oriented tobacco control policies, income redistribution and employment policies.

Health inequalities in Scotland are wider than in the rest of west and central Europe, and increasing on many measures [5]. The Scottish Government has stated that ‘reducing inequalities in health is critical to achieving the Scottish Government's aim of making Scotland a better, healthier place for everyone’ [6]. There is demand from policymakers and local planners for support in deciding which interventions are the most effective and cost-effective in reducing health inequalities. This reflects a gap in current scientific knowledge with important practical implications; although the broad principles about what works to reduce health inequalities have been articulated [7–8], the evidence about the likely magnitude of impact of specific interventions remains limited.

To address these gaps, tools have been developed to model impacts based on the most plausible available assumptions. For example, the Department of Health and London Public Health Observatory developed a tool which allows local authorities to estimate the effect of increased delivery of specific evidence based interventions on life expectancy [9]. The Scottish Public Health Observatory developed tools based on this work, initially modelling the impact of different investment scenarios in downstream interventions focussed on health behaviours (smoking, alcohol, obesity) [10]. The work described in this paper extends this modelling work to cover a broader range of interventions, using the best available data and evidence.

We aimed:

1. To quantify the impact of a range of public health interventions to reduce health inequalities in Scotland based on realistic scenarios for the delivery of ‘downstream’ interventions to individuals in deprived groups.
2. To compare ‘downstream’ interventions with universal, population-level approaches in terms of their impact on health inequalities.
3. To augment an existing suite of tools for informing decisions about how to reduce health inequalities in Scotland through the addition of further interventions and outcomes.
4. To provide decision-makers with comparisons of the effectiveness of differing strategies to tackle health inequalities.

## Materials and Methods

### Selection of interventions for modelling

We set out to examine interventions which included those addressing health behaviours and those addressing the broader socioeconomic determinants of health; and which varied in the degree to which they required individuals to ‘opt in’ (i.e. the degree of individual agency required). To do this we created a matrix using the determinants of health framework development by Dahlgren and Whitehead [11] against a dichotomous ‘population-wide’ or ‘individual’ axis. In their framework, Dahlgren and Whitehead describe the layers of influence on health. These range from individual characteristics that can impact on behaviours to promote or harm health (e.g. the role of gender in the decision to smoke or not) to structural factors in the outer layer, such as housing, employment, and access to services. The research team identified exemplar interventions for each layer and a project advisory group (PAG) composed of policy makers and public health professionals reviewed these and considered whether there were further interventions that could be added to ensure relevance. For each suggested intervention we conducted a rapid search for effect sizes for mortality and hospitalisation from reasonably valid and relevant studies using PubMed, Embase, Medline, CINAHL, and PsycINFO (S1 Table). English language studies conducted between 1992 and 2013 were identified and relevant reference lists were searched by hand to locate additional studies which met the eligibility criteria. Where necessary lead authors of original studies were contacted to provide further data of relevance to the model. Search terms included either the intervention (e.g. smoking cessation) or behaviour (e.g. alcohol consumption) or setting (housing) combined with: mortality; death; hospitalisation; hospital admission; all-cause; and inequality.

The PAG was then asked to approve a prioritised list of interventions on the basis of the desired spread of intervention type and the availability and quality of evidence of impact. Intensity levels of each intervention were informed by the available evidence and through consultation with the experts on our PAG. Some interventions were prioritised by the PAG even though there was an absence of a single defined intervention generating impacts along a theoretical causal chain. This process resulted in the following interventions being included:

1. Changes to taxation (a 1 pence increase in the basic rate of Scottish Income Tax and a 10% rise in the local Council Tax (a regressive property-based tax administered by local authorities)).
2. Changes to benefit payments (a 10% increase in the value of Jobseeker’s Allowance (JSA) (provided to those who can demonstrate they are actively seeking work) and Income Support (IS) (a benefit for those who are on low incomes), and a 10% increase in basic and 30-hour Working Tax Credits (reductions in tax liabilities for those on low incomes)).
3. Introduction of a minimum, or ‘living’ wage of £7.20 per hour (defined on the basis of the range of social security benefits available in c.2013).
4. A 10% increase in the level of tobacco taxation.
5. Greater provision of smoking cessation services.
6. Greater provision of alcohol brief interventions (ABIs).
7. Greater provision of the “Counterweight” [12] weight-management service
8. Changes in levels of employment.
9. Changes in the extent of active (walking and cycling) commuting to and from work.

We did not identify specific interventions for employment or active travel, and so we modelled the impact on admissions and mortality of specified changes in levels of employment and in levels of active travel to work occurring as a result of unspecified interventions. We were not able to include a housing intervention as originally planned because of the absence of evidence about impacts of housing improvements on mortality or hospitalisations [13].

### Overall approach to modelling

We created a model of the impact of a range of interventions on health and health inequalities. We compared the impact of the selected interventions on all-cause mortality and all-cause hospitalisations (and on inequalities in mortality and hospitalisations) to the counterfactual scenario of no intervention (for those interventions not currently being implemented), to the current level of intervention, or the current level of exposure to risk (for employment and active travel). We included adults aged 16 years or more for the smoking, tobacco tax, ABI and Counterweight models, those aged 15–69 years for the employment model, those aged 15–64 years for the active travel to work model and the whole population for the income models. We took a fixed (closed) cohort approach, and so did not model the impact on those not included in the original baseline cohort (i.e. immigrants and those reaching the minimum eligible age after the intervention started).

We obtained data from National Records of Scotland (NRS) on the current population by age, sex and quintile of the Scottish Index of Multiple Deprivation (SIMD), an area-based measure of material deprivation [14]. We estimated the number of people within each stratum who were exposed or unexposed to the relevant risk factor (e.g. smoking or unemployment) from the most appropriate data source (full details are provided elsewhere [15]).

Projected mortality rates obtained from NRS were used to estimate mortality rates by age, sex and calendar time. Historical data were used to estimate mortality and hospitalisation rates by age, sex, SIMD and calendar time. Parametric survival models (exponential distribution) were used to describe change in mortality and hospitalisation by age, sex, SIMD quintile and calendar time. The final function describing total mortality across all interventions combined projected rates for age, sex and calendar time from the projected data, with rates for SIMD from the historical data:

$${Mortality\;Rate=e}^{\left(18.19+0.082age+1.077sex(male)-0.014year+0.0095age*sex(male)+0.58SIMD1+0.2SIMD2-0.23SIMD4-0.55SIMD5\right)}$$

The hospitalisation rate function was based on historical data:

$${Hospitalisation\;Rate=e}^{(-6.06+0.025age-0.56sex(male)+0.0016year+0.01age*sex(male)+0.35SIMD1+0.14SIMD2-0.13SIMD4-0.25SIMD5})$$

where SIMD3 and female were reference categories in both cases.

Nonlinear Least Squares were used to produce a sigmoid function with two parameters and a single predictor (time) to describe the change in the rate ratio (i.e. the risk of mortality/hospitalisation for those exposed to the intervention compared to those not exposed) for each specific intervention over time. The income model was the exception for which the rate ratio was estimated using log-transformed standardised mortality and hospitalisation rates for Scotland on log transformed mean weekly equivalised household income after housing costs (data from the Institute of Fiscal Studies/Family Resources Survey) for each SIMD income domain quintile, using these coefficients to predict the effect of changing the income distribution (for mortality and hospitalisation separately). For each year of follow-up, we multiplied the mortality and hospitalisation rate by the rate ratio (between 0 and 1) for each intervention to provide an estimate of the effect of the intervention (delivered at time zero) during each year of follow-up. If, for example, the effect of an intervention on mortality was assumed to attenuate over time, the rate ratio tends to one over time. The difference in years of life lost has, for simplicity, been expressed as years of life gained (YLG). Where there were sufficient numbers to allow modelling, stratum-specific estimates were aggregated to calculate the impact of the policy on each locality and by SIMD quintile. The results are presented as the difference in the relative index of inequality (RII) (a regression-based index which summarises relative inequalities in health by socioeconomic status [16]) for mortality and hospitalisation between the population receiving the intervention and the population without an intervention. We also examined the effects of applying interventions equally across the population, in proportion to the ‘need’ across population quintiles, or of targeting them preferentially to the most deprived population quintile.

For YLL and hospitalisation we show absolute change and changes in relative inequalities accumulated over 10 and 20 years for the Scottish population. For interventions with a fixed delivery cost per intervention, we model an investment of an additional £5m; for active travel the population affected is set at 100,000; for employment we model 20,000 new jobs. The interventions with a fixed delivery cost were compared on a like-for-like basis (i.e. the impact of an additional £5m investment). Other interventions either had no net costs (e.g. increases to tobacco taxation) or no specific intervention or known cost (e.g. additional jobs).

Model specifications and sources of assumptions are detailed in S1 Table and S2 Table. More information on the methods, and the spreadsheet-based tools which allow the modification of the parameters in the model, are provided online [15] and in S1 Appendix.

### Income interventions

We identified only one source of estimates of changes in income distribution likely to result from tax and benefit changes in Scotland [17]. The change in risk associated with changed income distribution was applied to the whole population. We did not identify any studies estimating the direct impact of income changes on mortality or hospitalisation independent of wider economic changes. We therefore regressed log transformed standardised mortality and hospitalisation rates for Scotland (obtained from the Information Services Division (ISD) of NHS National Services Scotland, www.isdscotland.org/) on log transformed mean weekly equivalised household income after housing costs (data from the Institute of Fiscal Studies/Family Resources Survey) for each SIMD income domain quintile, using these coefficients to predict the effect of changing the income distribution. We did not attempt to estimate the direct costs or savings of the income interventions (i.e. the changes in tax revenues for government). The econometric model on which we were reliant did not estimate the potential effects of redistribution following changes to taxation, changes to public spending nor variation in the underlying assumptions.

### Tobacco tax

We modelled a 10% increase in tobacco product prices as a result of an increase to tobacco taxation. The population at risk (PAR) was defined as all Scottish residents aged ≥16 years who smoke (numbers estimated from the Scottish Household Survey (SHoS), 2012) and the exposure risk ratio was based on the risk of all-cause mortality and hospitalisation in individuals who currently smoke compared with those who have never smoked [18–20].

### Smoking cessation

The intervention was defined as an increase in the number of people offered the current mix of smoking cessation services provided by the Scottish NHS [21]. The PAR and exposure risk ratio were equivalent to those used in the tobacco tax model. The cost per smoking cessation intervention was estimated at £98 in 2011 [22]–this was updated to 2012 prices.

### Alcohol brief interventions (ABI)

The intervention was defined as an increase in the number of people offered the current Scottish ABI service. An alcohol brief intervention is a short, evidence-based, structured conversation about alcohol consumption that seeks to motivate and support an individual to change their drinking behaviour in order to reduce their consumption and risk of harm [23]. The PAR was defined as Scottish residents aged ≥16 years drinking hazardously or harmfully (Scottish Health Survey (SHeS), 2008–11 combined), assuming the non-intervention group would experience no change over time. The exposure risk ratio was based on the risk of all-cause mortality or hospitalisation in individuals who drink at hazardous and harmful levels in comparison to those who drink moderately [24–25]. The cost of an ABI was estimated at £25 in 2011 [26] and remained unchanged when inflated to 2012 prices after rounding.

### Counterweight

The Counterweight intervention was defined as an increase in the number of people offered the Counterweight weight management service; a structured programme available to patients with BMI >30 or >28 kg/m^2^ with an associated obesity-related disease [27]. The PAR was defined as Scottish residents aged ≥16 years with BMI ≥30 kg/m^2^ (SHeS, 2008–11 combined). The exposure risk ratio was based on the risk of all-cause mortality or hospitalisation in individuals who are obese compared to those who are not obese [28]. The cost of a Counterweight intervention was estimated at £72 in 2011 [15]–this was updated to 2012 prices.

### Employment

We identified no robust or generalisable evidence on the impact of specific interventions on employment. We therefore defined this model in terms of changes in employment levels rather than a specific intervention. We defined the population at risk (PAR) as Scottish residents aged 15–24 years not in full-time education, training or employment, or aged 25–69 years and not in employment. Our decision to focus on this group reflects the evidence on the mortality risks associated with not being in employment for those of working-age [29, 30] including for young adults not in full-time education [31]. It was decided to include adults up to 69 years old because there is some evidence that the protective effects of employment on mortality persist up to age 70 years [32]. Full-time students aged 15–24 years were excluded because of the protective effects of education on health among this group. The exposure risk ratio was based on the risk of all-cause mortality or hospitalisation in individuals who are employed compared to those who are not in employment. The cost per job created is assumed to be the £4,400 cited in Beatty et al [33], adjusted to 2012 prices.

### Active travel

The active travel intervention was defined as changes in commuting by walking and cycling rather than as a specific intervention. This choice was enforced by a lack of robust evidence on the impact of specific active travel interventions on mortality and hospitalisations. We assumed that the intervention would be structural (e.g. changing the physical environment or providing new infrastructure) and that changes in behaviour for the affected cohort would be sustained over time. We defined those at risk as the working population aged 16–64 years who commute to work in a car or van over distances of three miles or less, assuming that this would not change in the absence of an intervention.

The exposure risk ratio was based on the risk of all-cause mortality in individuals who are physically active (i.e. engage in at least 30 minutes of moderate intensity physical activity on most days of the week) compared to those who are inactive [34]. We did not attempt to estimate the direct cost of the active travel intervention. Estimates of the impact of increased physical activity on all-cause hospitalisations were unavailable.

### Hospitalisation costs

Geue et al. [35] estimated the average cost of a continuous inpatient stay between 2001 and 2007 at £2,113 based on a mean (SD) number of admissions of 15,576 (34.1)—this was adjusted to 2012/13 prices.

### Sensitivity analysis

We conducted the following sensitivity analyses:

#### Income

For income models, the estimated changes in income resulting from each specific intervention were drawn from published results that were based on a household level model of the Scottish economy which included a behavioural response element [36]. It was not possible to identify appropriate parameters from published results to enable sensitivity analysis of the impact of the policy levers concerned on income inequality. Sensitivity analysis was therefore confined to the assumption that the relationship between income and mortality was not confounded. For illustrative purposes, it was arbitrarily assumed that confounding attenuated the impact of income changes on mortality by 25% and 50%, giving more conservative estimates of the impact of the interventions.

#### Tobacco tax/smoking cessation

Using base population data ‘elements’ of smoking prevalence; leavers (i.e. people who quit 1–2 years ago), starters (including those ≥16 years and occasional/regular smoking 15-year-olds) and population estimates, the Scottish Government has produced projections of smoking prevalence to 2045. Two projected smoking prevalence models are presented; a basic model where none of the elements changes from baseline, and an enhanced model where assumptions are made regarding changes in each element (e.g. decreases in prevalence among 15-year-olds and those aged ≥16 years and increasing proportions of successful quitters). The tobacco models use the basic smoking prevalence projection estimates. The sensitivity analysis used the enhanced model estimates of prevalence change between 2012 and 2032 [37].

#### Alcohol Brief Interventions

Given the complexities in estimating future prevalence of hazardous and harmful drinking, the ABI model assumes that the PAR in the untreated group will remain static over the 20-year period. A sensitivity analysis was carried out assuming a 10% increase or decrease in the untreated PAR over the 20-year period.

The 65% compliance rate used in the ABI model from the review by Kaner [38] and colleagues is likely to be unduly optimistic in the Scottish context, where implementation of ABIs has been in different settings and population groups from the original trials. The sensitivity analyses therefore include much more conservative (15%) or extreme (100%) compliance with the intervention.

#### Counterweight

Given the difficulties of projecting obesity levels we based our assumptions on US trends. USA data on obesity levels amongst adults suggest that Scotland is around 10–15 years behind the USA in its obesity trends [39]. The prevalence of obesity in Scotland in 2003 (22.9%) was similar to USA levels in 1991 (23.2%). The data suggest that obesity levels in the USA will reach 52% by 2030. If the trend in Scotland followed that in the US (applying a 12-year time lag alongside obesity prevalence data from SHeS), and assuming no additional effective obesity prevention in Scotland, obesity levels for the 16–64 year age group could reach 41% by 2030, an increase of 58% over 2008 levels. Adjusted to reflect levels in the population aged ≥16 years, the projected prevalence is 43%. This estimate equates to an increase in the prevalence of obesity in Scotland of around 14.5% over the next 20 years, a figure which is used in the Counterweight model. Given the uncertainty surrounding this estimate, we considered more conservative increases in obesity prevalence (half of this, 7.25%) or a scenario where the projected obesity prevalence increases return to zero over a five-year period.

The compliance rate is defined as the proportion of individuals enrolled into Counterweight who attended a 12-month follow-up appointment: approximately 40%. The average weight loss of 3.7kg, (1.36 kg/m^2^) derived from experience of Counterweight implementation, is applicable to this group only. Although the published figure for compliance is 28%, this included a large number of participants from a Health Board where follow-up rates were particularly low. Excluding this Health Board generates an estimate of about 40% and this is considered more in line with what is achievable in routine practice in Scotland. We considered the impact of more conservative (28%) or more extreme (100%) compliance with the intervention.

## Results

### Years of life lost

**Fig 1:**
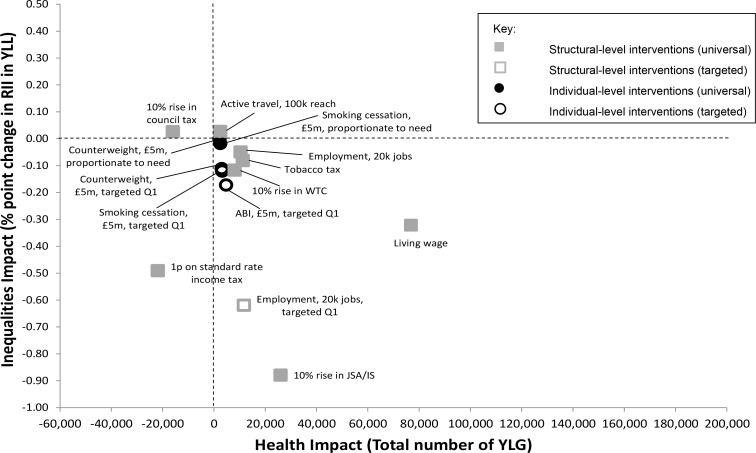
Modelled changes after 10 years in the RII of cumulative years of life lost and the total number of years of life gained for all the modelled interventions (based on £5m investment where associated costs are estimated).

Most of the modelled interventions reduced both YLL and relative inequalities after 10 years, but with varying effects (Fig 1). Introduction of a living wage generated the largest beneficial impact on YLL, and led to a modest reduction in health inequalities (with a gain of 77,000 years of life and a decrease of 0.32 percentage points in the RII over 10 years). A 10% increase in JSA/IS had a less prominent beneficial impact on YLL, but a greater impact on health inequalities (a reduction of 26,000 in YLL and a decrease of 0.88 percentage points in the RII over 10 years). Increases in employment had a more modest impact on YLL and only reduced inequalities when targeted to the most deprived groups, while tobacco taxation and a 10% increase in working tax credit improved YLL but had minimal impact on health inequalities. In contrast, increases in active travel to work had minimally positive effects on YLL but marginally widened health inequalities. Increasing council tax worsened both YLL and health inequalities and a 1 pence increase in the standard rate of income tax (SRIT) reduced inequalities modestly but worsened YLL. ABIs, smoking cessation and Counterweight had only minimal impacts on YLL and health inequalities, even when exclusively targeted to the most deprived areas.

**Fig 2:**
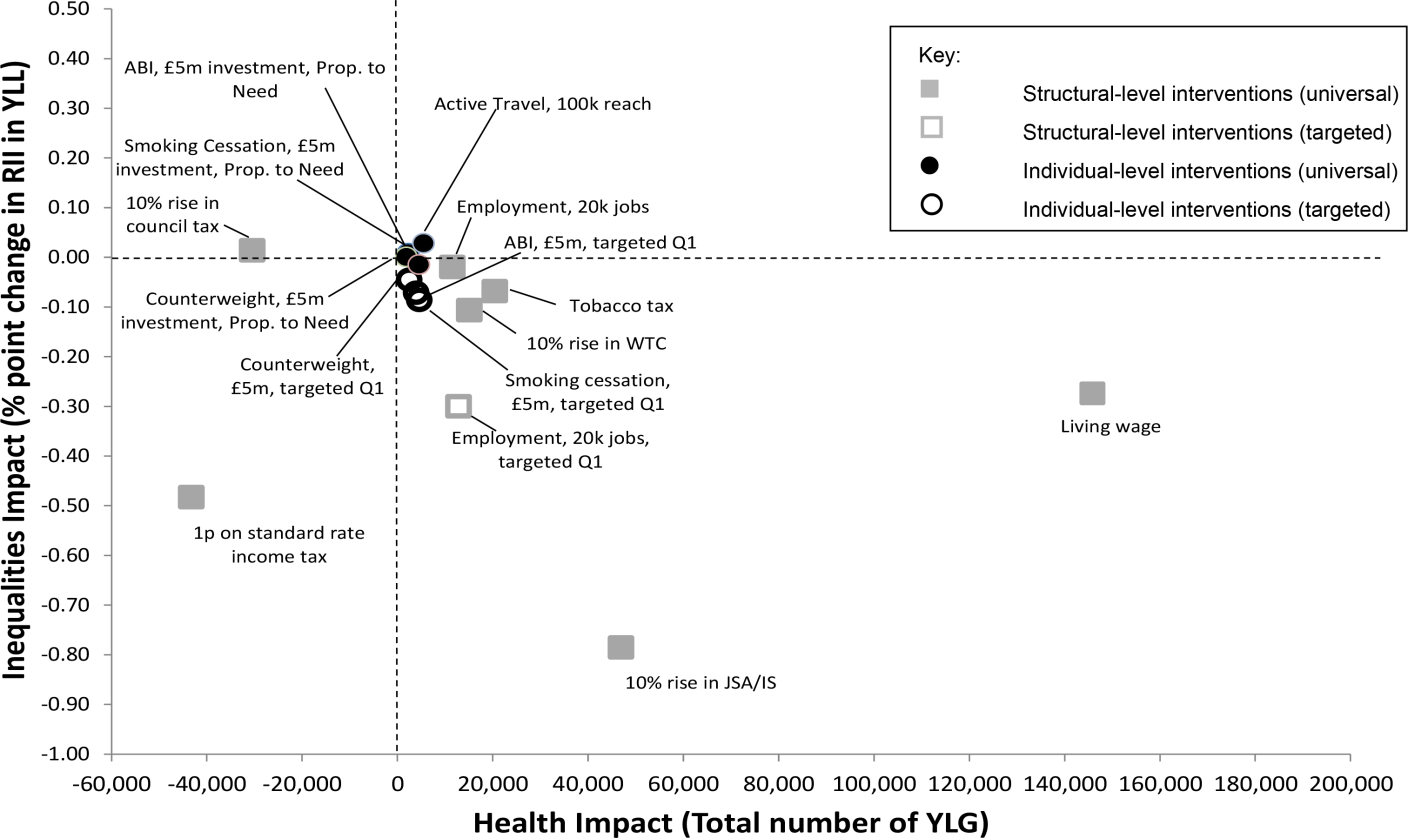
Modelled changes after 20 years in the RII of cumulative years of life lost and the total number of years of life gained for all the modelled interventions (based on £5m investment where associated costs are estimated).

After 20 years the protective impact of most of the interventions on YLL and inequalities continued to accumulate, although there were some notable exceptions (Fig 2). For example, in both the Counterweight and the ABI models, the protective impact of the interventions reduced over time.

### Hospitalisations

**Fig 3:**
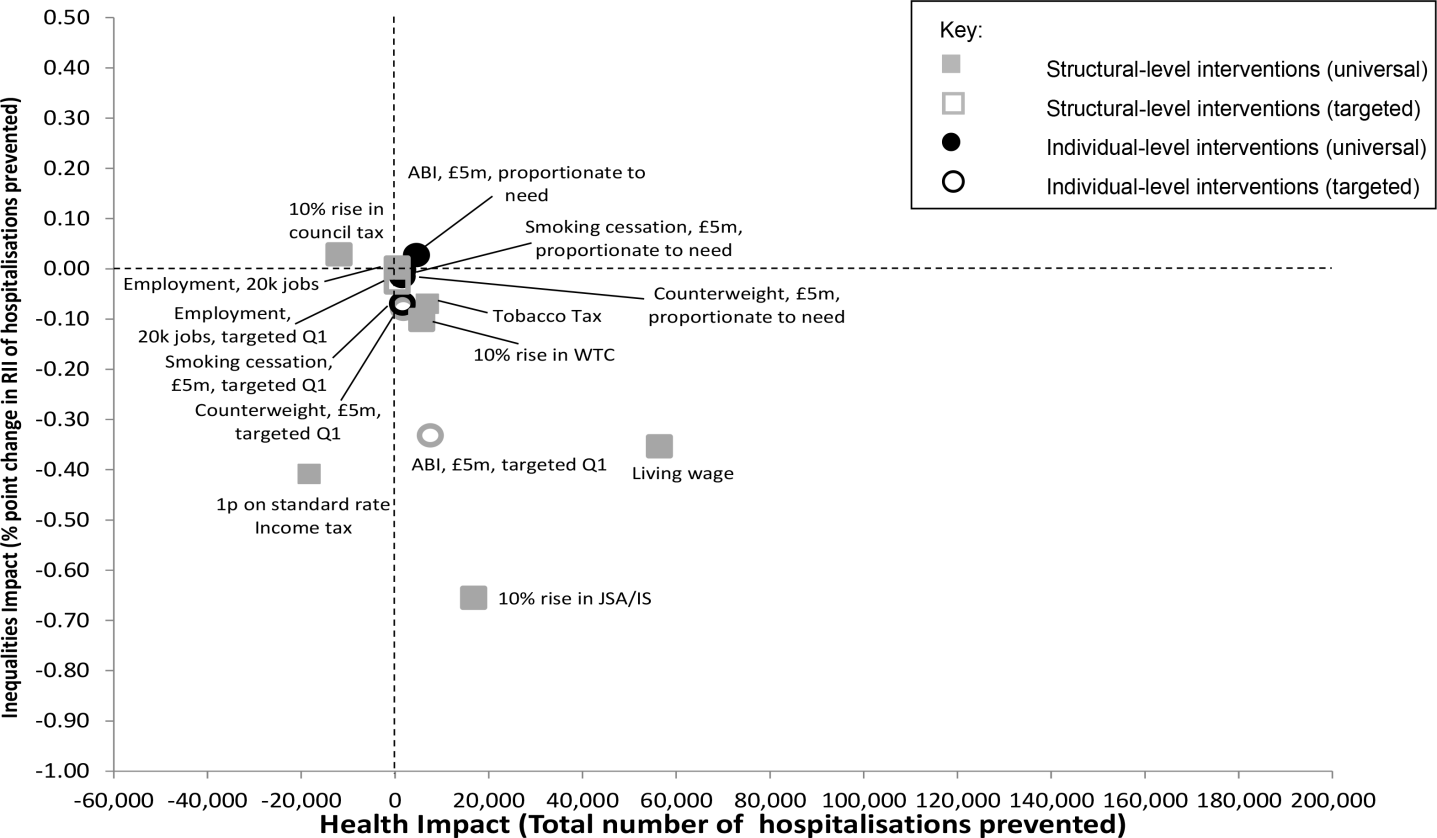
Modelled changes after 10 years in the RII of cumulative continuous inpatient stays and the total number of hospitalisations prevented for all the modelled interventions (based on £5m investment where associated costs are estimated).

Most interventions achieved an absolute reduction in the number of hospitalisations and in inequalities after 10 years, again with differing effects (Fig 3). Introduction of a living wage generated the largest beneficial impact, and led to a modest reduction in health inequalities (with more than 56,000 hospitalisations prevented and a decrease of 0.35 percentage points in RII over 10 years). A 10% increase in JSA/IS had a less prominent beneficial impact on hospitalisations, but a larger impact on health inequalities (17,000 hospitalisations prevented/decrease of 0.66 percentage points in RII over 10 years). Alcohol brief interventions had modestly positive impacts on hospitalisations and health inequalities only when strongly socially targeted, while tobacco taxation and a 10% rise in working tax credit also modestly reduced hospitalisations but had minimal impact on health inequalities. Increasing council tax negatively impacted on hospitalisations and health inequalities and a 1 pence increase in the SRIT reduced inequalities modestly but increased hospitalisations. The remaining interventions had small beneficial impacts on both outcomes or made no difference.

**Fig 4:**
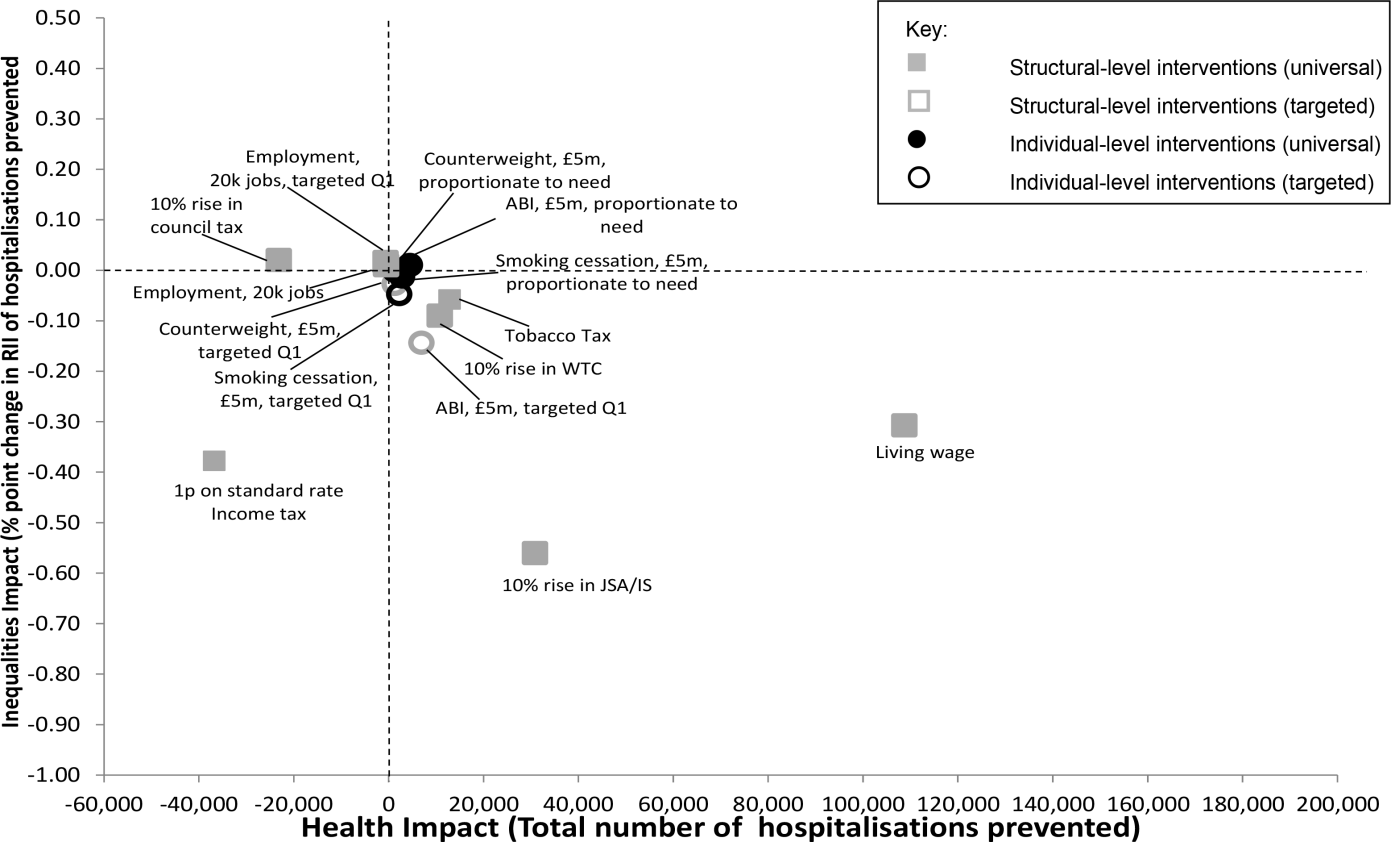
Modelled changes after 20 years in the RII of cumulative continuous inpatient stays and the total number of hospitalisations prevented for all the modelled interventions (based on £5m investment where associated costs are estimated).

After 20 years, the protective impact of most of the interventions on hospitalisations and inequalities continued to accumulate, although there were some notable exceptions (Fig 4). A similar fall in impact over time was observed within the Counterweight and ABI models. In addition, the protective impact of gaining employment also declined over time to the point where, after 20 years, hospitalisations began to increase for the cohort involved.

### Financial impact

The model facilitates estimation of direct healthcare savings, in a notional sense, from prevented hospitalisations (i.e. the value of inpatient days potentially freed up rather than actual savings) (Table 1). For example, over 10 years, introduction of a living wage would save £138m, a 10% JSA/IS would save £41m and a 1 pence increase in prices as a result of tobacco taxation would save £17m. Conversely, a 10p rise in council tax and adding 1 pence on to the standard rate of income tax would incur additional costs of £29m and £45m respectively (this does not include the money raised with the increases in tax which would offset this, nor the impacts of using that money for redistribution or increases in public spending).

**Table 1:** Estimated direct financial savings from prevented hospitalisations 10-years after implementation in proportion to need.

Intervention	(£m) Whole population	(£m) Targeted to the most deprived quintile
Introduction of a living wage	138.0	32.4
10% rise in JSA/IS	41.1	36.7
Tobacco taxation	17.0	5.8
10% rise in Working Tax Credit	14.0	4.6
ABIs (£5m investment)	11.2	2.4
Counterweight (£5m investment)	4.0	1.0
Smoking cessation (£5m investment)	3.7	1.3
Employment, 20,000 jobs	1.2	0.3
10% rise in council tax	-29.2	-7.6
1 pence on standard rate of income tax	-44.6	-3.0

### Sensitivity analysis summary

Applying the enhanced model estimates of projected prevalence of smoking over the next 20 years to the tobacco models results had a minimally positive impact on reported outcomes (results not shown). In addition, sensitivity analyses using different prevalence projections for obesity and hazardous/harmful drinking produced only small changes to outcomes, with scenarios where prevalence is projected to fall over the next 20 years producing the most positive results.

The largest impact on results was seen when adjustments were made to the compliance rates for the Counterweight and ABI models (results not shown). As one might expect, higher levels of compliance with the intervention impacted positively on health outcomes, particularly for preventing hospitalisations via ABIs. Moreover, increasing levels of compliance with Counterweight and ABIs only reduced health inequalities when socially targeted, although much more effectively through ABIs in the hospitalisations model than in the mortality model.

As expected, sensitivity analysis showed that if confounding attenuated the relationship between income and mortality by 50%, the estimated impact of the intervention was reduced by the equivalent extent for all outcomes. For example, the percentage change in RII as a result of introducing a living wage would be reduced from -0.32 to -0.16.

We carried out additional analyses to show the intensity of intervention required to match the magnitude of results achieved for the most positive outcomes, assuming that 100% of those eligible for treatment could be reached through each intervention. For the absolute reductions in mortality and hospitalisations, the benchmark intervention selected was the Living Wage, while for the relative reductions in mortality the benchmark intervention was increasing the value of JSA/IS by 10%. Results were compared after 10 years. In S3 Table, we show that it would be theoretically possible to achieve a level of impact on years of life saved similar to introducing the Living Wage if an intervention was available that helped 147,000 people find work. None of the other interventions was able to achieve similar results. The nearest individually-based approach was smoking cessation, which could avoid almost 37,000 years of life lost, but only if 100% of smokers in Scotland were recruited to smoking cessation programmes. For hospitalisations, none of the other interventions came close to matching the impact of introducing the Living Wage, though if 100% of smokers were recruited to smoking cessation programmes this would result in almost 23,000 fewer hospitalisations after 10 years.

In terms of reducing inequalities in mortality, it would be possible to achieve similar results to increasing the value of JSA/IS by 10% if an intervention was available to increase employment by 345,000 (close to the theoretical maximum of the eligible population at risk). None of the other interventions was able to achieve similar results (S4 Table). For reducing inequalities in hospitalisations, none of the other interventions was able to achieve comparable results to increasing the value of out of work benefits. The closest results were seen for increasing the standard rate of income tax by 1 pence, followed by introducing a Living Wage.

## Discussion

### Summary of the main results

Introduction of a living wage generated the largest beneficial impact on health, and led to a modest reduction in health inequalities. Increases to benefits had modest beneficial impacts on health and health inequalities. Income Tax increases had a negative impact on population health but reduced inequalities, while council tax increases worsened both health and health inequalities (based on the non-redistributive models available). Increases in active travel to work had minimally positive effects on health, but widened health inequalities. Increases in employment reduced inequalities only when targeted to the most deprived groups. Tobacco taxation had modestly positive impacts on health but little impact on health inequalities. Alcohol brief interventions had modestly positive impacts on health and health inequalities only when strongly socially targeted, while smoking cessation and Counterweight weight-reduction programmes had only minimal impacts on health and health inequalities even when socially targeted. The choice of intensity we selected for interventions is to some extent arbitrary and illustrative; the accompanying online tool (http://www.scotpho.org.uk/comparative-health/health-inequalities-tools/intervention-tools/informing-investment-to-reduce-health-inequalities-iii) allows users to examine the effect of varying intensities (S2 Appendix). It would be possible to legitimately compare different intensities of intervention for some of the interventions which do not have known fixed costs (e.g. employment, active travel, tobacco tax) and so the comparison involving these interventions need to be interpreted in the knowledge that a more or less intensive intervention may cost approximately the same as the fixed costs interventions (smoking cessation, ABIs and counterweight). These results reflect model specifications and need careful interpretation.

### Interpreting the main results

The income interventions involve modest changes to the income distribution, include a number of conservative assumptions relating to behavioural responses and do not include the impacts of the changes on public spending or the wider economy. For example, the changes in council and income tax have opposite impacts on inequalities because of their respective regressive and progressive nature. However, neither improves overall health, because they reduce the incomes of individuals as there is no capacity for using the additional revenue (e.g. for redistribution or providing services) in those models. Obviously if increased tax revenues were used to finance effective interventions, or to increase the incomes of the lowest income groups, this could improve population health and reduce inequalities.

Interventions involving specific health behaviours (tobacco tax, smoking cessation, active travel, Counterweight and ABIs) are based on the assumption that the change in exposure to risk resulting from the intervention leads to the changes in outcomes seen in observational studies. This is likely to overestimate the impact of the interventions. The inequalities impact of ABIs, and to a lesser extent smoking cessation and an increase in tobacco tax, may be underestimated in the models because they rely on self-reported health behaviours.

There are challenges to comparing the interventions on a strictly like-for-like basis, as the sensitivity analyses making comparisons with the most ‘positive’ interventions demonstrate. It would be difficult, and perhaps implausible, to recruit 100% of those eligible for any of the downstream (smoking cessation, ABI, Counterweight) interventions. Fiscal and political issues, as well as practical difficulties, also make it unlikely that the employment and active commuting models could reach 100% of their population at risk. Nevertheless, what these comparisons suggest is that while downstream interventions make a positive contribution to improving population health and (for smoking cessation and counterweight, on assumptions shown here) reducing health inequalities, they could not plausibly generate impacts comparable to more upstream interventions, even using relatively extreme assumptions.

### Strengths and limitations of the modelling

Modelling offers a ‘flexible, cost-effective, evidence-based research method with the capacity to inform public health policymakers regarding the implementation of population health interventions to reduce social inequalities in health’ [40]. These models provide a novel means of comparing the long-term impacts of a range of interventions. They utilise the best available evidence relevant to the Scottish context and mark an improvement in the support available to decision-makers when allocating resources and planning interventions and policies. Although the modelling requires a number of assumptions, these are explicit and can be refined as better evidence becomes available. Sensitivity analyses allow uncertainty around the estimates to be made explicit.

The approach is limited by the relatively small number of interventions included (in particular, the income interventions available looked at only minor changes in the income distribution). This could inadvertently divert attention from worthwhile but under-studied interventions, biasing attention to health behaviour interventions that are easier and cheaper to study [41]. We could not identify specific interventions for active travel and employment, which necessitated using changes in physical activity and employment as proxies.

Adopting a rapid, rather than systematic, approach to reviewing the literature for relevant studies may have led to the omission of a small number of relevant studies. Despite this, our use of an expert advisory group and contact with a wide range of experts in each field reassures us that such omissions are likely to have been minimal.

To ensure that measures were comparable across interventions we restricted the analysis to two outcomes (mortality and all-cause hospitalisations). Data were not available to examine a broader range of health measures (including wellbeing and positive health).

The models do not include impacts beyond the original cohort. This creates an ageing cohort and, as expected, a decline in mortality inequalities [42] in populations unaffected by the interventions. This is compounded by the projected reduction in mortality used in the NRS analyses (which itself is uncertain [43]). Some interventions may have an impact on populations unexposed to the risk factor of interest (e.g. non-smokers may be prevented from starting smoking through an increase in tobacco tax). For interventions with such preventive potential our approach may underestimate the overall impact, but of the interventions we studied, this is only likely to apply to tobacco taxation. Limited evidence about differential impacts across population strata meant that we had to assume the risk ratios applied evenly across groups for all of the models except those involving income interventions (which may have biased the results of the inequalities analyses in an unknown direction).

Differences in inequalities over time are measured using the 2012 version of SIMD (based on 2011 data); however it is possible that, as a result of the interventions applied, the relative position of some areas in terms of deprivation may change. This is not taken account of in the analysis, which treats the people as a fixed cohort living in areas whose relative deprivation within Scotland is also considered fixed.

In some models (e.g. employment) we used longitudinal studies with repeated measures of exposures and health outcomes over time to estimate the risk factor -outcome relationship. In others we used comparisons of outcomes where only baseline assessments of exposure (e.g. alcohol, BMI) were available. The tobacco tax, smoking cessation, ABI and counterweight models focused on ‘downstream’ exposures (e.g. health behaviours), and derived effect sizes from observational studies which did not examine a change in the exposure. These models are vulnerable to overestimation of impact on inequalities because we assume that a change in exposure at that distal point in the causal chain will improve outcomes despite there being several other causal pathways through which more ‘upstream’ exposures such as poverty will continue to generate mortality [2] [44–47] [48].

The income model was limited by the small number of interventions for which new income distributions were available. Income model impacts were considered to be immediate and constant over time and allowed for changes by SIMD. The available interventions achieved only very limited reductions in income inequality and did not extend to some of the more radical options that have been proposed recently within the Scottish Parliament [49–51]. As noted above, the impact of income interventions is unlikely to be subject to bias because of other competing exposures and so the relative positive impact compared to the other interventions is likely to be underestimated. While there is a theoretical possibility of reverse causality in the relationship between income and health, in practice this has been shown to be minimal [52]. We also had to approximate area-based income deprivation quintiles with household income quintiles and this is likely to have underestimated the impact of the intervention because a large number of people will have been misclassified into an incorrect quintile [53].

The impacts in the employment model may be underestimated because it assumes that some of the non-intervention group gain employment each year and that the protective impact of gaining employment declines over time. The long-run increase in hospital admissions in the intervention group in the employment model is due to the limited impact of employment on hospitalisation (in contrast to mortality), combined with an larger but ageing population of survivors who accrue more admissions. It is also possible that there may be other specific employment interventions which we were not able to model which have differing impacts on health and health inequalities.

The observed increase in the impact of the tobacco interventions over time is due to the long-term effects of stopping smoking. For ABIs and Counterweight, the models assume the protective impact is likely to be short-term. The resulting postponed deaths create a ‘delayed mortality’ effect which eventually reduces their long term impact on health and inequalities. The time frame over which these interventions are compared is therefore crucial in determining which is more likely to be favourable.

Those included in the SHeS sample have been shown to be substantially healthier than the general population [54], and their reported alcohol intake much lower than the amount of alcohol sold in Scotland [55–56]. As a result, the inequality impact of the interventions (such as ABIs and smoking interventions) which use self-reported health behaviours to estimate the PAR, is likely to be underestimated. Despite these caveats, ABIs do appear to impact on inequalities more than the other downstream interventions, albeit at modest levels. This is because both excessive alcohol consumption and alcohol-related harm are strongly associated with deprivation, to a greater extent than smoking or obesity related harm.

We only examined the impacts of active travel on those already in work and who own a car or van (both less prevalent in the most deprived). This explains the modelled increase in health inequalities. In reality, the impact on health inequalities is less certain since structural changes in the environment would also impact on non-commuting travel, reduce road traffic injuries (more common in deprived areas), and improve air quality. There may be other specific active travel interventions which we have not considered that may have different impacts on health and health inequalities.

A final limitation of this study is it does not attempt to incorporate the full direct costs or benefits of the interventions. We have not included estimates of the direct economic benefits of ‘years of life saved’, since these were not readily available for all-cause mortality for Scotland. On the other hands, we have also excluded from consideration the direct fiscal costs of upstream interventions. This could be complex to assess since while the downstream, lifestyle interventions are typically constant and ‘one-off’ investments, the tax, spend and employment costs would be recurring (albeit changing over time as the economy and labour market changed). Incorporating these elements was beyond the scope of this study, but could be considered in future iterations of the model.

### Targeting

The model demonstrates the importance of targeting strategies in tackling health inequalities. The extent to which policies should be implemented universally or targeted at specific groups depends on the nature of the health problem, its context and the potential effectiveness and efficiency of the solution [57]. With reference to Geoffrey Rose’s population health strategy [58], Benach and colleagues [57] present a typology of four policy scenarios to address health inequalities (i.e., targeted and health gap, universal policy with additional focus on gap, ‘redistributive policy’, and ‘proportionate universalism’ or universal policy with increasing benefits through the gradient). Table 2 applies this typology to the interventions we considered.

**Table 2:** Typology of four policy scenarios of health inequalities reduction, classified by focus of reduction and extent of benefits, with examples from modelled interventions.

Benefits to social groups:	Inequality reduction focus	
	Gap	Gradient
*Selective*	1. Targeted interventions on worst-off only	3. Redistributive policy
	- 10% ↑JSA/IS	- 10% ↑Council Tax
		- 1 pence SRIT
		- Tobacco taxation
*Universal*	2. Universal policy with additional focus on gap	4. Proportionate universalism
	- Smoking cessation	- Alcohol brief interventions
	- Counterweight	- Active travel
	- Employment	- 10% ↑ WTC
		- Living wage

The feasibility and desirability of targeting all interventions to the most deprived groups is debatable. Targeting has requirements that often do not exist in practice. For example, only 34% of Scottish low- income households are in the 20% most deprived areas in Scotland [53]. Targeting may result in interventions being seen as ‘poor people’s services’, creating stigma, undermining quality and undermining the collectivism which is essential to support the funding of public services [59].

One approach to avoiding the dangers of ‘means testing’ is to create services which are both universal and proportionate to need [60]. For example, the national performance management target in Scotland requires universal smoking cessation services to achieve at least 80,000 successful quits (one month post-quit) including 48,000 in the 40% most-deprived areas over the three years ending March 2014 [61].

## Conclusions

These models help decision-makers understand the likely inequality impacts of investment in, or of disinvestment from, various interventions. Currently, resource allocation decisions are often made in the absence of such evidence. Our approach allows the assumptions and baseline conditions of the models to be changed, the impacts of interventions to be compared, impacts to be modelled locally and investment and direct hospitalisation costs to be compared.

We developed approaches that used the best available data and evidence to estimate reductions in hospitalisations, YLL and health inequalities associated with a range of public health interventions. We were able to develop a transparent and usable interactive tool that allows users to model a range of interventions designed to reduce health inequalities.

Interventions have markedly different effects on mortality, hospitalisations and inequalities. The most effective (and likely cost-effective) interventions for reducing inequalities were regulatory and tax options which affect income. Interventions focused on individual agency were much less likely to impact on inequalities, even when targeted at those in the most deprived communities. In broad terms, these results fit with previous evidence that interventions that tackle inequalities in the socio-economic environment and regulatory interventions are more likely to reduce health inequalities, while those requiring individual agency are less effective [7].

## Supporting Information

S1 Appendix Informing Investment to Reduce Health Inequalities Toolkit and Longer-term Follow-up: Description of Approach. (DOCX)

S2 Appendix Permission from the Scottish Public Health Observatory to republish information. (DOCX)

S1 Table Exposure risk ratios and proportion of Population at Risk (PAR) eligible for treatment for each model. (DOCX)

S2 Table Summary of evidence and assumptions used for intervention impact. (DOCX)

S3 Table Level of intervention required to match absolute impact of introduction of Living Wage, up to 100% recruitment of eligible population. (DOCX)

S4 Table Level of intervention required to match impact on inequalities of 10% increase in JSA/IS, up to 100% recruitment of eligible population. (DOCX)
