# Supplementary material for: Informing Investment to Reduce Inequalities: A Modelling Approach
Source: PLoS One. 2016 Aug 3;11(8):e0159256. doi: 10.1371/journal.pone.0159256 (PMC4972318; doi:10.1371/journal.pone.0159256)
Supplement: S1 Appendix — (DOCX) [file pone.0159256.s001.docx]

**Appendix A: Informing Investment to Reduce Health Inequalities Toolkit and Longer-term Follow-up: Description of Approach**

# Aims

The purpose of the Informing Investment to Reduce Health Inequalities toolkit is to allow policy-makers to estimate the effect of particular policies on mortality and hospitalisation over time according to inequalities. The policies can relate to interventions across the determinants of health (including ‘upstream’, ‘downstream’, individually-focussed and population-wide interventions).

The toolkit allows users to create policy scenarios by assigning:-

- the total number of interventions in a population (e.g. 10,000 alcohol brief interventions)
- the distribution of interventions by Scottish Index of Multiple Deprivation (SIMD) quintile (e.g. increased interventions delivered to people living in more deprived areas)
- the timing of interventions are delivered (e.g. during years 1, 2, 3, 4 of follow-up). This is currently set to year 1 for all tools.

The toolkit then produces estimates of the effect of a policy on mortality and hospitalisations at 2, 10 and 20 years after baseline:-

- for the entire population
- for sub-groups defined on the basis of age, sex and SIMD quintile

This report explains the epidemiological basis, implementation, and underlying assumptions which apply to all the interventions included in the toolkit. Additional details for specific interventions are given in the main report.

# Estimating mortality

For clarity, we use smoking cessation as a practical example throughout this technical guide. We also use the term “treated” for people who were the subject of an intervention, even where that intervention (as in the case of altering income through changes in taxation and benefits) is applied to a whole society rather than an individual.

In order to estimate the impact on mortality of a given policy compared to the counterfactual scenario of not implementing that policy, we need to calculate the total number of deaths over the period of follow-up (e.g. 2 years, 5 years 10 years) under both the policy and the counterfactual. The total number of deaths will include deaths in people who are unexposed to a risk factor (e.g. non-smokers), people who are exposed but untreated (hereafter untreated, e.g. smokers not given smoking cessation advice) and people who are both exposed and treated (hereafter treated, e.g. smokers who are given smoking cessation advice).

The total number of deaths over follow-up time in a closed cohort is the cumulative incidence of mortality (e.g. if there are 50 deaths over 5 years, then 50 is the 5-year cumulative incidence). When cumulative incidence is divided by the number of people alive at baseline then this proportion is termed the cumulative incidence rate.

Over very short time periods, the cumulative incidence can be reasonably approximated by the mortality rate (λ) times the follow-up time (t). Over longer time periods, however, the following equation is needed to calculate the cumulative incidence rate from the mortality rate ((Rothman et al. 2008, Page 43):-

| 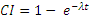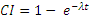 | Equation [1] |
| --- | --- |

where CI is the cumulative incidence rate, e is a mathematical constant (approximately 2.72), λ is the instantaneous (or current) mortality rate, and t is the follow-up time.
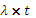

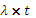
 is the area under the curve of mortality rate over time, where mortality rate is constant (or is assumed to be constant).

| Figure 1 Constant mortality rate | Figure 2 Variable mortality rate |
| --- | --- |
|  |  |

If, instead, the mortality rate varies across time as in Figure 2 (which is likely given that mortality rates generally increase with age) then the area under the curve is calculated via integration rather than by multiplication:-

| 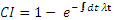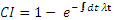 | Equation [2] |
| --- | --- |

The implication of equation 2, is that if we can describe the mortality rate of a group within the population over time using a mathematical function, we can estimate the total number of deaths (cumulative incidence x number in group at baseline) for that group by integrating the function, and transforming the integral as in Equation [2].

As such, for any follow-up time period, we can sum the cumulative incidence for mortality for people who are unexposed, treated and untreated for both the policy and the counterfactual scenarios as:-

| 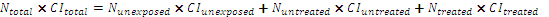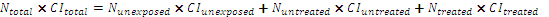 | Equation [3] |
| --- | --- |

The cumulative incidence rates for the groups unexposed, untreated and treated are the same under the policy and counterfactual. In each case, N refers to the number of people alive at baseline. The number in the treated and untreated groups will differ under the policy and counterfactual scenarios. Hence the difference in the cumulative incidence can be calculated as:-

| 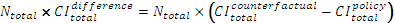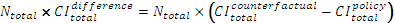 ) | Equation [4] |
| --- | --- |

The cumulative incidence rate can also be used to calculate the number of years of life lost.

| 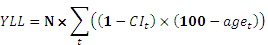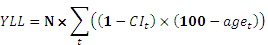 | Equation [5] |
| --- | --- |

Where t is the follow-up time in years, YLL is the Years of Life Lost, CI is the Cumulative Incidence at time t and age is age at the follow-up time t. YLL is essentially a sum of the total number of deaths weighted by the age at death, with deaths at younger ages having a higher weight.

## Summary statistics for cumulative and YLL

These statistics, the CI and the YLL for the policy and the counterfactual scenarios, can be calculated for specific age, sex and SIMD quintile strata within the population. In each population there are approximately 160 strata (16 age-bands, 2 sexes and 5 SIMD quintiles). The difference in CI and YLL for the policy and counterfactual can be summed for the whole population, or for sub-groups within the population formed by aggregating across age, sex and/or SIMD strata. By aggregating into sub-groups the impact of a policy can be compared across different sections of the population, for example the reduction in cumulative incidence among the least and most deprived quintiles can be compared.

# Estimating mortality rates

A crucial first step to calculating the cumulative incidence for each group is to obtain a mathematical function which describes mortality rate across age, sex, SIMD and calendar time for unexposed, untreated and treated individuals. We now describe how we did so.

## Total mortality rate

The mortality rate in the unexposed, untreated and treated are all derived from the projected mortality rate for the total Scottish population. We used estimated mortality rates for years 2011 to 2035 (from the 2010 based sub-national population projections) by age, sex and calendar year from National Records of Scotland (NRS). Using regression modelling we obtained a formula which describes the change in mortality over time according to age, sex and calendar time. Coefficients for age, sex and calendar time were obtained (intercept=18.188, age=0.082, sex(male)=1.077, year=-0.014, age*sex(male)=0.0095). This formula fits the projected mortality data well (*Appendix 1*).

Unfortunately, NRS do not produce separate estimates for each SIMD quintile. Therefore, using data from 2000 to 2010 we examined the association between SIMD and mortality rate. We found that the relative (multiplicative) effect of SIMD was consistent across age, sex and calendar time (*Appendix 1*). Consequently we assumed, for the counterfactual case where no new policy is instituted, that the same association will hold over 20 years of follow-up. As such, using historical data we modelled total mortality according to age, sex, calendar time, and SIMD and obtained coefficients for SIMD (SIMD1=0.577, SIMD2=0.199, SIMD3=0, SIMD4=-0.229, SIMD5=-0.547). We then combined the age, sex and calendar time coefficients for the projected mortality rates from NRS with SIMD coefficients from historical data to obtain a formula describing projected total mortality according to age, sex, calendar time and SIMD. SIMD 3 was the reference value.

This resulting function describes total mortality across all of the interventions.

| 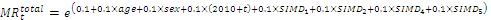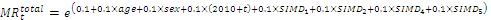 | Equation [6] |
| --- | --- |

## Mortality rate in untreated (exposed)

Since the total mortality rate is the sum of the mortality rate in people who are exposed and unexposed weighted by the proportion exposed and unexposed, and since the mortality rate in the exposed divided by the mortality rate in the unexposed is the rate ratio for the exposure, the following equation relates mortality rate in people exposed to a given risk factor (e.g. smoking) to total mortality:-

| 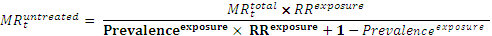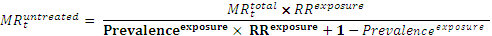 | Equation [7] |
| --- | --- |

where MR is the mortality rate in the untreated (but exposed) at time t, RR_exposure_ is the rate ratio for the exposure and P_exposure_ is the prevalence of the exposure in the population. Since the mortality in exposed people depends on the prevalence of the exposure and the rate ratio for the exposure, the mortality in the unexposed and exposed (untreated) is specific to each toolkit. The rate ratio for each exposure was obtained from the published literature. We assumed that the rate ratio for the exposure was identical for all age, sex and SIMD strata for which the cumulative incidence was estimated. (The income model provides special case in both of these respects; due to limitations in the available literature routine Scottish data were used to calculate rate ratios, and analysis was done for each SIMD quintile).

## Mortality rate in unexposed

The mortality rate in the unexposed group is the mortality rate in the untreated (but exposed) group divided by the rate ratio for the exposure.

| 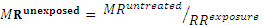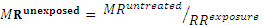 | Equation [8] |
| --- | --- |

## Mortality rate in treated group

The mortality rate in the treated group is the product of the mortality rate in the untreated group times the rate ratio for the intervention. Unlike the rate ratio for the exposure, the rate ratio for the intervention was allowed to vary across time. For the income toolkit, different rate ratios were applied to each SIMD quintile to reflect the fact that we wished to model increasing income in some quintiles, and decreasing income for other quintiles. For all other toolkits the rate ratio for the intervention (i.e. the efficacy of the intervention) was assumed to be constant across age, sex and SIMD quintile.

| 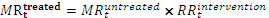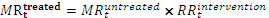 | Equation [9] |
| --- | --- |

With the exception of the income model, the rate ratio for the intervention was obtained from the published literature. For the income model the rate ratio was estimated using log-transformed standardised mortality and hospitalisation rates for Scotland (data obtained from Information Services Division (ISD) of NHS National Services Scotland, <http://www.isdscotland.org/>) on log transformed mean weekly equivalised household income after housing costs (data from the Institute of Fiscal Studies/ Family Resources Survey) for each SIMD income domain quintile, using these coefficients to predict the effect of changing the income distribution. Where it was available, we used published evidence to obtain estimates of the change in efficacy over follow-up time. Often, however, it was necessary to assume the pattern of change in efficacy over time.

Figure 3 provides a hypothetical example of a rate ratio with high efficacy at time zero (0.65), whose efficacy diminished towards 1 over twenty years of follow-up. Figure 4 shows how the instantaneous mortality rate in the treated group would be modified (over time) by the action of this intervention. We chose functions which adequately described the change in rate ratio over time according to the published evidence and our assumptions of the likely change in efficacy over time (refer to chapters on specific interventions). As such, the form for the mathematical function (in this case sigmoid) was determined by the available evidence and epidemiological assumptions. The method used to obtain the coefficients for the functions are given in appendix 2.

| Figure 3: Rate ratio over time | Figure 4: Mortality rate |
| --- | --- |
|  |  |

# Assumptions

In summary, therefore, we calculated total mortality and total mortality weighted by age (termed Years of Life Lost) using the following assumptions:-

1. After a policy is applied the population is fixed, although mortality rates do increase with ageing. The toolkit does not take into account inward movement into the population from births, or the effects of immigration. This means that the toolkit can only be used to estimate the absolute impact on the health of people in whom the policy is implemented. Alternatively, if only the difference in mortality and hospitalisation with the policy scenario compared to the counterfactual scenario is needed, the weaker assumption that the policy will not affect birth rates or immigration is sufficient.
2. That projected mortality rates by age, sex, SIMD and calendar year will be accurate over the next 20 years
3. That the mortality rates can be approximated using an exponential formula
4. That SIMD will have the same association with mortality over the next 20 years as it has over the past 10 years (under the counterfactual scenario of no policy change)
5. That the rate ratio for the exposure is constant across age, sex, SIMD quintile and calendar time
6. That the rate ratio for the intervention is constant across age, sex and (except for the intervention on income) SIMD
7. Assumptions used to estimate the proportion of the population exposed to each risk factor are provided within the specific documentation for each intervention

Assumption 3 has been tested graphically (*Appendix 1*) and it can be seen that the function fits the data well. If the function did not fit the data well a more complex function with additional parameters could have been used instead.

# Hospitalisation

Unlike death, hospitalisation can recur. Therefore, the total count of hospitalisation can be calculated as the hospitalisation rate times the person time. Person time is the time people are at risk of an event, and is the sum across persons of the at risk time for each individual. For our analysis, the at-risk time is the time from the start of follow-up until death. Deaths in the policy and counterfactual scenario will differ; hence the total person time will differ between the policy and counterfactual scenario. The following equation calculates the person time during each year of follow-up and multiplies that by the current hospitalisation rate to obtain the hospitalisation count:-

| 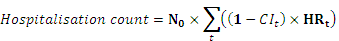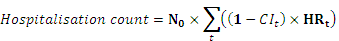 | Equation [10] |
| --- | --- |

where N_0_ is the number of people within an age, sex and SIMD stratum at baseline, CI_t_ is the cumulative incidence at time t and HR_t_ is the hospitalisation rate at time t. 1-CI_t_ times N_0_ is the person time at time (t) and the sum of 1-CI_t_ times N_0_ is the total person time.

As for mortality rate, the hospitalisation rate in the unexposed, untreated and treated was calculated from the total hospitalisation rate, the prevalence of the exposure, the rate ratio for the exposure and the rate ratio for the intervention.

Unlike for mortality we did not have projections for hospitalisation rates and used historical SMR01 rates held within ISD for all estimates. Whereas mortality rates showed a clear downward trend over time, hospitalisation rates have showed only a very small change over the past 10 years, conditional on age, sex and SIMD quintile. Although the model assumes that this modest decline in hospitalisation will continue, the degree of uncertainty around this assumption is greater than for mortality.

As for cumulative incidence and years of life lost for mortality, hospitalisation under the policy and counterfactual scenario can be summed for the population, or can be summed across sub-groups within the population defined on the basis of age, sex and SIMD.

In addition to the assumptions for mortality, this approach for calculating hospitalisations assumes that hospitalisation rates are distributed homogenously across person time (conditional on age, sex, SIMD quintile and calendar time).

## Implementation

The approach to calculate mortality and hospitalisation described above was implemented as follows:-

1. In R, an open source statistical package:
   1. parametric survival models (exponential distribution) were used to obtain a formula to describe total mortality/hospitalisations according to age, sex, SIMD quintile and calendar time (for mortality and for hospitalisation)
   2. non-linear models were used to obtain coefficients for a formula which describes change in the rate ratio for the intervention over time (for mortality and hospitalisation) (Appendix 2)
   3. the mortality rate in the untreated (exposed) was calculated using Equation [7]
2. Using published data from the relevant data sources (e.g. Scottish Health Survey) and data from NRS we tabulated the number of people within each age, sex and SIMD stratum and the proportion of those exposed to each risk factor (e.g. smoking) in Excel (For the income model, we assumed 100% of the population was affected by the intervention).
3. Using built-in Excel functions we designed the spreadsheets to allow users of the toolkit to choose how many people should be “treated” under a given policy during each year of follow-up, across how many years the number of treatment should be applied (currently set to all occurring in year 1 for all tools), and how these treatments should be distributed across SIMD quintiles.
4. In Visual Basic for Applications (VBA, Office’s built-in programming language which is designed to allow users to add functionality to Office programs) using the coefficients obtained from 1, we wrote functions which:
   1. Describe change in the rate ratio for the intervention over follow-up time
   2. Describe the change in mortality rate across age, sex, SIMD quintile and follow-up time for people who are unexposed, untreated and (also using (a)) treated
   3. Calculate the area under the curve for b
   4. Calculate the cumulative incidence from c
   5. Calculate the years of life lost from d
   6. Describe the rate ratio for the intervention over time for hospitalisation
   7. Describe the change in hospitalisation rate across age, sex, SIMD quintile and follow-up time for people who are unexposed, untreated and (also using (f)) treated
   8. Calculate the hospitalisation count from d and g
5. These functions (4) were used within excel to calculate the cumulative incidence, years of life lost and hospitalisation count for each age, sex and SIMD stratum, for each follow-up time of interest, under both the policy and the counterfactual scenarios.
6. The stratum-specific estimates from (5) were aggregated to calculate the impact of the policy on whole populations and sub-groups.
7. The functionality in 4 was replicated in R.

Detail on the specifics can be found in the documentation embedded in the VBA and R code itself (Appendices 1-3).

**References**

Rothman KJ, Greenland S, Lash TL. Modern Epidemiology. 3rd ed. Lippincott Williams & Wilkins; 2008.

# Appendix 1 Modelling mortality and hospitalisation rates

We obtained functions which described the mortality data using the Survival package in R. The data were modelled using the exponential and Weibull distributions. As we were not seeking to make inferences or extrapolate beyond the data the model checking was largely done visually by checking the fitted values against the rates. The standard errors and P-values from these models were used neither in the model selection nor in the final toolkit.

The annotated code used to run the models are provided below.

### R code to create formulas describing mortality rates and hospitalisation rates. These formulas are used in the R code

# "Calculates_CI_YLL_and_hospitalisation_in_R.R" and in the VBA toolkit

########### This code uses parametric survival models (ie not Cox) to produce functions to describe mortality and hospitalisation

# according to year, age, gender and SIMD. An interaction term for age and gender improved the fit a lot for hospitalisation

# and a little for mortality. No other interactions improved the fit

# Exponential distribution fits as well as Weibull so have chosen to use exponential as it is simpler

# Over fitting is not an issue as we are not seeking to extrapolate from these data to any other data

# only to represent the rates in a function for conciseness

# This R code, when run, will produce

# 1. plot of the rates as points with the fitted model as a line

# 2. an Rdata file with the coefficients

# 3. a CSV file with the coefficients

# The required inputs are

# 1. A .csv file with mortality rates in the correct format

# 2. A .csv file with the hospitalisation rates in the correct format

# packages

# install.packages (c("survival", "ggplot2")) # If not already installed need to uncomment this line

library (survival)

library (ggplot2)

library (reshape2)

# Set working directory - this should be where .csv files are stored and where R will save the output

# setwd("\\\\Stats\\phip\\Projects\\Health Inequality Tool\\HITS III\\LongTermTrends\\Final_Model\\")

# Read files

mort <- read.csv (file = "MortalityQuintiles.csv", as.is = TRUE)

mort.proj <- read.csv (file = "Mort_proj_1035.csv", as.is = TRUE)

names(mort.proj) <- names(mort)

hosp <- read.csv ("HospAdmQuintiles0112.csv", as.is = TRUE)

#hosp.proj <- read.csv (" " , as.is = TRUE)

# Transform data, written as function as the same code is applied to the hospitalisation and mortality data

MortHospClean <- function (mydata, type, simd.present = TRUE) {

names(mydata) <- c("year", "gender", "simd", "agegroup", "rate")

# create age as a numeric variable and drop agegroup

mydata$age <- as.numeric (substr(mydata$agegroup,1,2) ) + 2.5

mydata$agegroup <- NULL

# change SIMD to factor, not much change in fit, but easier to set 3 to reference

mydata$simd <- factor (mydata$simd)

if (simd.present == TRUE) mydata$simd <- relevel (mydata$simd, 3)

if (simd.present == FALSE) mydata$simd <- NULL

# select age range desired

mydata <- subset (mydata, age >=15 & age<80)

# make event variable for model to run

mydata$event <- 1

# convert rate into per person year, not per 1000 person years for mort, and not per 1000 person years for hospitalisation

if (type == "mort") mydata$rate <- mydata$rate /1000

if (type == "hosp") mydata$rate <- mydata$rate /1000

mydata

}

# apply function to transform each dataset, additional argument for projections

mort <- MortHospClean (mort, type = "mort")

mort.proj <- MortHospClean (mort.proj, type = "mort", simd.present = FALSE)

hosp <- MortHospClean (hosp, type = "hosp")

#hosp.proj <- MortHospClean (hosp.proj, type = "hosp", simd.present = FALSE)

# Run regression model based on event rates for mortality and hospitalisation

# Exponential distribution

model.surv.e <- survreg (Surv(time = rate, event) ~ age + simd + gender + year + gender:age, dist = "exponential",data = mort)

model.surv.e.hosp <- survreg (Surv(time = rate, event) ~ age + simd + gender + year + gender:age, dist = "exponential",data = hosp)

model.surv.e.proj <- survreg (Surv(time = rate, event) ~ age + gender + year + gender:age, dist = "exponential",data = mort.proj)

#model.surv.e.proj.hosp <- survreg (Surv(time = rate, event) ~ age + gender + year + gender:age, dist = "exponential",data = hosp.proj)

# Weibull distribution

model.surv.w <- update (model.surv.e, dist = "weibull")

model.surv.w.hosp <- update (model.surv.e.hosp, dist = "weibull")

model.surv.w.proj <- update (model.surv.e.proj, dist = "weibull")

#model.surv.w.proj.hosp <- update (model.surv.e.proj.hosp, dist = "weibull")

# Show model results

summary(model.surv.e)

summary(model.surv.w)

summary(model.surv.e.proj)

summary(model.surv.w.proj)

summary(model.surv.e.hosp)

summary(model.surv.w.hosp)

# summary(model.surv.e.proj.hosp)

# summary(model.surv.w.proj.hosp)

# Produce predicted rates from the model for the plots used to examine the fit

mort$weib <- predict (model.surv.w)

mort$expo <- predict (model.surv.e)

mort.proj$weib <- predict (model.surv.w.proj)

mort.proj$expo <- predict (model.surv.e.proj)

hosp$weib <- predict (model.surv.w.hosp)

hosp$expo <- predict (model.surv.e.hosp)

# Create plots of rates and predicted rates for men and women, for mortality and hospitalisation

# Red lines are for an exponential distribution, blue for Weibull

plot.surv.men <- ggplot (subset (mort, gender == "Male"), aes (x = age, y = rate)) + geom_point() +

facet_grid (simd~year) +

geom_line (aes(x = age, y = expo), colour = "red") +

geom_line (aes(x = age, y = weib), colour = "blue") +

ggtitle ("Men, mortality")

plot.surv.women <- ggplot (subset (mort, gender == "Female"), aes (x = age, y = rate)) + geom_point() +

facet_grid (simd~year) +

geom_line (aes(x = age, y = expo), colour = "red") +

geom_line (aes(x = age, y = weib), colour = "blue")+

ggtitle ("Women, mortality")

plot.surv.men.proj <- ggplot (subset (mort.proj, gender == "Male"), aes (x = age, y = rate)) + geom_point() +

facet_wrap (~year) +

geom_line (aes(x = age, y = expo), colour = "red") +

geom_line (aes(x = age, y = weib), colour = "blue") +

ggtitle ("Men, mortality, projected")

plot.surv.women.proj <- ggplot (subset (mort.proj, gender == "Female"), aes (x = age, y = rate)) + geom_point() +

facet_wrap (~year) +

geom_line (aes(x = age, y = expo), colour = "red") +

geom_line (aes(x = age, y = weib), colour = "blue")+

ggtitle ("Women, mortality, projected")

plot.surv.men.hosp <- ggplot (subset (hosp, gender == "Male"), aes (x = age, y = rate)) + geom_point() +

facet_grid (simd~year) +

geom_line (aes(x = age, y = expo), colour = "red") +

geom_line (aes(x = age, y = weib), colour = "blue") +

ggtitle ("Men, hospitalisation")

plot.surv.women.hosp <- ggplot (subset (hosp, gender == "Female"), aes (x = age, y = rate)) + geom_point() +

facet_grid (simd~year) +

geom_line (aes(x = age, y = expo), colour = "red") +

geom_line (aes(x = age, y = weib), colour = "blue")+

ggtitle ("Women, hospitalisation")

# Save plots as a PDF with today's date

mydate <- format (Sys.Date(), "%Y%m%d")

filename <- paste ("Fit using data from Jan 2014, hosp and mort (historical and projected), interaction age_gender, run on ", mydate, ".pdf", sep = "")

pdf (filename)

plot.surv.men + theme_gray (base_size =8)

plot.surv.women + theme_gray (base_size =8)

plot.surv.men.proj + theme_gray (base_size =8)

plot.surv.women.proj + theme_gray (base_size =8)

plot.surv.men.hosp + theme_gray (base_size =8)

plot.surv.women.hosp + theme_gray (base_size =8)

dev.off()

# Create coefficients table and save as CSV file and save as both sets of coefficients as objects in .Rdata

mortality.coefficients <- coef (model.surv.e)

hospitalisation.coefficients <- coef(model.surv.e.hosp)

mortality.coefficients.projected <- c(coef(model.surv.e.proj), simd1 = NA, simd2 = NA, simd4 = NA, simd5 = NA)

#hospitalisation.coefficients.projected <- c(coef(model.surv.e.proj.hosp), simd1 = NA, simd2 = NA, simd4 = NA, simd5 = NA)

mortality.coefficients.projected <- mortality.coefficients.projected [ names(mortality.coefficients)]

#hospitalisation.coefficients.projected <- hospitalisation.coefficients.projected [ names(mortality.coefficients)]

# coefficients for age, sex and age:sex interaction very similar for projected and historical data

# not too dissimilar for year, quite big difference in intercepts

all.coefficients <- rbind (mortality.coefficients, mortality.coefficients.projected, hospitalisation.coefficients)#, hospitalisation.coefficients.projected)

filename <- paste ("Coefficients using data from Jan 2014, hosp and mort, interaction age_gender, run on ", mydate, ".csv", sep = "")

write.csv (all.coefficients, file = filename)

filename <- paste ("Coefficients using data from Jan 2014, hosp and mort (historical and projected), interaction age_gender, run on ", mydate, ".Rdata", sep = "")

save (mortality.coefficients, mortality.coefficients.projected, hospitalisation.coefficients, file = filename)

Figure 5: Fitted predictions for mortality and hospitalisation

Dots represent rate data (from GROS and ISD), lines represent fitted values, the blue line is for the Weibull model and red line is for the exponential model. For brevity, only male data is shown.

#


# Appendix 2 Modelling change in rate ratios

The following R code shows how a non-linear model was used to produce a function which was used to represent the change in rate ratio over time.

### R code to create formulas describing rate ratios for death and hospitalisation. These formulas are used in the R code

# "Calculates_CI_YLL_and_hospitalisation_in_R.R" and in the VBA toolkit

# This code uses Nonlinear Least Squares to produce a function describing the change in rate ratio

# This approach constrains the rate ratio to lie between 0 and 1

# If the rate ratio is zero, you need to take it's inverse to calculate the formula, and then

# invert the function when running it in the "Calculates_CI_YLL_and_hospitalisation_in_R.R" code (invert.rr <- 1)

# and/or in the VBA toolkit (invert_rr = 1).

# The particular form of the non-linear model is a sigmoid function with two parameters and a single predictor, time.

# This function is paramaterised below as intuitively as possible, in terms of the gain and threshold.

# The threshold is the point on the x-axis which corresponds to y = 0.5 (which can of course lie outside the range of values we are interested in)

# The gain is how steeply the curve moves through that point

# You may need to supply reasonable values as starting points for the gain and threshold if the plots show that the estimates

# don't fit the values well

# Inputs

# a .csv file with time and rate ratios for hospitalisation and death

# starting values for the gain and threshold, if model won’t fit with the defaults

# outputs

# model coefficients (a .csv file)

# plots of the rate ratio and the rate ratio described as a function and two example plots

# read in file

intervention <- read.csv ("employment_rate_ratios.csv")

intervention.name <- "employment"

# Sigmoid function with two parameters

mort <- nls(rr.mort ~ 1 / (1 + exp(-gain * (time - threshold))), data=intervention, start=list(gain=1, threshold = 0))

intervention$rr.mort.predicted <- predict(mort)

hosp <- nls(rr.hosp ~ 1 / (1 + exp(-gain * (time - threshold))), data=intervention, start=list(gain=0.2, threshold = -5))

intervention$rr.hosp.predicted <- predict(hosp)

# Plot points to formula

pdf (paste (intervention.name, ".pdf", sep = ""))

par (mfrow = c(2,2))

plot (0:20, intervention$rr.mort, ylim = c(0,1), main = "Mortality rate ratio", xlab = "Time (years)", ylab = "Rate ratio")

lines (0:20, predict(mort))

plot (0:20, intervention$rr.hosp, ylim = c(0,1), main = "Hospitalisation rate ratio", xlab = "Time (years)", ylab = "Rate ratio")

lines (0:20, predict(hosp))

# Produce example plots

rr <- function (time, gain, threshold) {1 / (1 + exp(-gain * (time-threshold)))}

plot (0:20, rr(0:20,1, 10), ylim = c(0,1), main = "eg: gain = 1, thresholds = 5, 10 & 15", xlab = "Time (years)", ylab = "Rate ratio", type = "l")

lines (0:20, rr(0:20,1, 5))

lines (0:20, rr(0:20,1, 15))

plot (0:20, rr(0:20,0.1, 10), ylim = c(0,1), main = "eg: threshold = 10, gain = 0.1, 0.2 and 0.5", xlab = "Time (years)", ylab = "Rate ratio", type = "l")

lines (0:20, rr(0:20,0.2, 10))

lines (0:20, rr(0:20,0.5, 10))

dev.off()

# save coefficients

rr.hosp.coef <- coef (hosp)

rr.mort.coef <- coef (mort)

write.csv (rbind (rr.mort.coef, rr.hosp.coef), file = paste (intervention.name, ".csv", ""))

**Intervention rate ratios over time for mortality**

| \| **Year** \| **Smoking Cessation** \| **ABIs** \| **Counterweight** \| **Tobacco tax** \| **Employment** \| **Active Travel** \| \| --- \| --- \| --- \| --- \| --- \| --- \| --- \| \| 1 \| 0.9803 \| 0.972 \| 0.964 \| 0.9944 \| 0.674 \| 0.987 \| \| 2 \| 0.9862 \| 0.977 \| 0.971 \| 0.9961 \| 0.781 \| 0.987 \| \| 3 \| 0.9862 \| 0.981 \| 0.978 \| 0.9960 \| 0.812 \| 0.987 \| \| 4 \| 0.9861 \| 0.986 \| 0.985 \| 0.9960 \| 0.825 \| 0.987 \| \| 5 \| 0.9861 \| 0.991 \| 0.992 \| 0.9960 \| 0.843 \| 0.987 \| \| 6 \| 0.9860 \| 0.995 \| 1 \| 0.9960 \| 0.860 \| 0.987 \| \| 7 \| 0.9860 \| 1 \| 1 \| 0.9960 \| 0.877 \| 0.987 \| \| 8 \| 0.9860 \| 1 \| 1 \| 0.9960 \| 0.894 \| 0.987 \| \| 9 \| 0.9859 \| 1 \| 1 \| 0.9960 \| 0.910 \| 0.987 \| \| 10 \| 0.9859 \| 1 \| 1 \| 0.9960 \| 0.924 \| 0.987 \| \| 11 \| 0.9858 \| 1 \| 1 \| 0.9960 \| 0.930 \| 0.987 \| \| 12 \| 0.9858 \| 1 \| 1 \| 0.9959 \| 0.937 \| 0.987 \| \| 13 \| 0.9858 \| 1 \| 1 \| 0.9959 \| 0.943 \| 0.987 \| \| 14 \| 0.9857 \| 1 \| 1 \| 0.9959 \| 0.948 \| 0.987 \| \| 15 \| 0.9857 \| 1 \| 1 \| 0.9959 \| 0.954 \| 0.987 \| \| 16 \| 0.9857 \| 1 \| 1 \| 0.9959 \| 0.956 \| 0.987 \| \| 17 \| 0.9857 \| 1 \| 1 \| 0.9959 \| 0.959 \| 0.987 \| \| 18 \| 0.9856 \| 1 \| 1 \| 0.9959 \| 0.961 \| 0.987 \| \| 19 \| 0.9856 \| 1 \| 1 \| 0.9959 \| 0.964 \| 0.987 \| \| 20 \| 0.9856 \| 1 \| 1 \| 0.9959 \| 0.966 \| 0.987 \| |  |  |  |  |  |  |
| --- | --- | --- | --- | --- | --- | --- | --- | --- | --- | --- | --- | --- | --- | --- | --- | --- | --- | --- | --- | --- | --- | --- | --- | --- | --- | --- | --- | --- | --- | --- | --- | --- | --- | --- | --- | --- | --- | --- | --- | --- | --- | --- | --- | --- | --- | --- | --- | --- | --- | --- | --- | --- | --- | --- | --- | --- | --- | --- | --- | --- | --- | --- | --- | --- | --- | --- | --- | --- | --- | --- | --- | --- | --- | --- | --- | --- | --- | --- | --- | --- | --- | --- | --- | --- | --- | --- | --- | --- | --- | --- | --- | --- | --- | --- | --- | --- | --- | --- | --- | --- | --- | --- | --- | --- | --- | --- | --- | --- | --- | --- | --- | --- | --- | --- | --- | --- | --- | --- | --- | --- | --- | --- | --- | --- | --- | --- | --- | --- | --- | --- | --- | --- | --- | --- | --- | --- | --- | --- | --- | --- | --- | --- | --- | --- | --- | --- | --- | --- | --- | --- | --- | --- | --- |

**Income**

|  | SIMD1 (most deprived) | SIMD2 | SIMD3 | SIMD4 | SIMD5 (least deprived) |
| --- | --- | --- | --- | --- | --- |
| (Living Wage) | 0.99 | 0.9902 | 0.9889 | 0.9937 | 0.9977 |
| (10% increase WTC (basic and 30-hour amounts)) | 1 | 0.9986 | 0.9991 | 0.9998 | 1.0000 |
| (10% rise council tax) | 1 | 1.0016 | 1.0020 | 1.0016 | 1.0011 |
| (10% increase JSA/IS) | 0.99 | 0.9991 | 0.9998 | 1.0000 | 1.0000 |
| (1p on SRIT) | 1 | 1.0013 | 1.0026 | 1.0038 | 1.0052 |

**Intervention rate ratios over time for hospitalisations:**

| \| **Year** \| **Smoking Cessation** \| **ABIs** \| **Counterweight** \| **Tobacco tax** \| **Employment** \| **Active Travel** \| \| --- \| --- \| --- \| --- \| --- \| --- \| --- \| \| 1 \| 0.9838 \| 0.983 \| 0.972 \| 0.9954 \| 0.965 \| N/A \| \| 2 \| 0.9886 \| 0.986 \| 0.977 \| 0.9968 \| 0.979 \| N/A \| \| 3 \| 0.9886 \| 0.989 \| 0.983 \| 0.9967 \| 0.974 \| N/A \| \| 4 \| 0.9886 \| 0.992 \| 0.988 \| 0.9967 \| 0.976 \| N/A \| \| 5 \| 0.9886 \| 0.994 \| 0.994 \| 0.9967 \| 0.979 \| N/A \| \| 6 \| 0.9885 \| 0.997 \| 1 \| 0.9967 \| 0.982 \| N/A \| \| 7 \| 0.9885 \| 1 \| 1 \| 0.9967 \| 0.984 \| N/A \| \| 8 \| 0.9885 \| 1 \| 1 \| 0.9967 \| 0.986 \| N/A \| \| 9 \| 0.9884 \| 1 \| 1 \| 0.9967 \| 0.989 \| N/A \| \| 10 \| 0.9884 \| 1 \| 1 \| 0.9967 \| 0.990 \| N/A \| \| 11 \| 0.9884 \| 1 \| 1 \| 0.9967 \| 0.989 \| N/A \| \| 12 \| 0.9884 \| 1 \| 1 \| 0.9967 \| 0.989 \| N/A \| \| 13 \| 0.9884 \| 1 \| 1 \| 0.9967 \| 0.988 \| N/A \| \| 14 \| 0.9883 \| 1 \| 1 \| 0.9967 \| 0.988 \| N/A \| \| 15 \| 0.9883 \| 1 \| 1 \| 0.9967 \| 0.988 \| N/A \| \| 16 \| 0.9883 \| 1 \| 1 \| 0.9967 \| 0.988 \| N/A \| \| 17 \| 0.9883 \| 1 \| 1 \| 0.9967 \| 0.989 \| N/A \| \| 18 \| 0.9883 \| 1 \| 1 \| 0.9966 \| 0.989 \| N/A \| \| 19 \| 0.9883 \| 1 \| 1 \| 0.9966 \| 0.990 \| N/A \| \| 20 \| 0.9882 \| 1 \| 1 \| 0.9966 \| 0.990 \| N/A \| |  |  |  |  |  |  |
| --- | --- | --- | --- | --- | --- | --- | --- | --- | --- | --- | --- | --- | --- | --- | --- | --- | --- | --- | --- | --- | --- | --- | --- | --- | --- | --- | --- | --- | --- | --- | --- | --- | --- | --- | --- | --- | --- | --- | --- | --- | --- | --- | --- | --- | --- | --- | --- | --- | --- | --- | --- | --- | --- | --- | --- | --- | --- | --- | --- | --- | --- | --- | --- | --- | --- | --- | --- | --- | --- | --- | --- | --- | --- | --- | --- | --- | --- | --- | --- | --- | --- | --- | --- | --- | --- | --- | --- | --- | --- | --- | --- | --- | --- | --- | --- | --- | --- | --- | --- | --- | --- | --- | --- | --- | --- | --- | --- | --- | --- | --- | --- | --- | --- | --- | --- | --- | --- | --- | --- | --- | --- | --- | --- | --- | --- | --- | --- | --- | --- | --- | --- | --- | --- | --- | --- | --- | --- | --- | --- | --- | --- | --- | --- | --- | --- | --- | --- | --- | --- | --- | --- | --- | --- |
|  |  |  |  |  |  |  |

**Income:**

|  | SIMD1 (most deprived) | SIMD2 | SIMD3 | SIMD4 | SIMD5 (least deprived) |
| --- | --- | --- | --- | --- | --- |
| (Living Wage) | 0.99 | 0.9923 | 0.9914 | 0.9951 | 0.9982 |
| (10% increase WTC (basic and 30-hour amounts)) | 1 | 0.9989 | 0.9993 | 0.9999 | 1.0000 |
| (10% rise council tax) | 1 | 1.0012 | 1.0015 | 1.0012 | 1.0008 |
| (10% increase JSA/IS) | 0.99 | 0.9993 | 0.9999 | 1.0000 | 1.0000 |
| (1p on SRIT) | 1 | 1.0010 | 1.0020 | 1.0029 | 1.0040 |

# Appendix 3 VBA code

The following VBA code details the calculations for cumulative incidence, years of life lost and hospitalisations prevented over time, using Tobacco Tax as an example.

'# For this documentation we will refer to:-

'# 1. users (users who only feel comfortable changing excel inputs)

'# 2. modifiers (users who feel comfortable editing VBA code)

'# 3. designers (those who designed the toolkit)

'# In Excel, functions written in VBA are described as "user-defined" functions, this is potentially confusing, so we will call them bespoke functions.

'# In R we largely follow Google'#s R style guide, where dot.notation is used for objects and CamelBackNotation is used for functions.

'# In excel we use underscores instead of .s as these are not allowed in VBA, while capitalisation is under the control of the VBA editor.

'# This R code produces the same results and mimics the approach used in the VBA code

'# All of the R functions are bespoke VBA functions - eg rr, mortality.rate, mortality.rate.rr, rr.h, hosp.rate, hosp.rate.h, CumulativeIncidence etc

'# Some bespoke VBA functions are written as snippets of code in R which use for (1 in n) style loops

'# TECHINCAL NOTE - R software designers wrote R with vector manipulations in mind whereas VBA was not,

'# hence for consistency we use a lot of loops in R, when it would be more conventional to use R'#s vector functionality

'# Excel allows users to change values, these are described as "cell-values" here.

'# We will distinguish between back-end cell values (eg number of people in a particular stratum) and

'# front-end cell values (eg number of people treated)

'# VBA also allows users to enter "constants" in the VBA code. These can be changed only by opening up and modifying the VBA code, hence

'# these "constants" can only be changed by modifiers and designers.

'# Inputs ----

'# mortality rate parameters - see "Create_Mortality_hospitalisation_formula.R" - as constants

'# rate ratio parameters - see "Create_rate_ratio_formula.R" - as constants

'# Age, sex, SIMD quintile, number exposed etc - as back-end cell-values

'# Number treated, year intervention follow-up starts etc - as front-end cell-values

'# Outputs ----

'# table of cumulative incidence, hospitalisation courts and YLL for policy and baseline interventions

'''''''code starts here

Option Explicit

'#### Constants ----

'# 1a. parameters for the mortality function - modifiers (not users) could change these value

Const Intercept As Double = 18.1877130850229

Const age_coef As Double = 8.19275291014469E-02

Const simd1 As Double = 0.576547965287156

Const simd2 As Double = 0.199455277946668

Const simd3 As Double = 0

Const simd4 As Double = -0.228807765165563

Const simd5 As Double = -0.546955533913082

Const gender_female = 0

Const gender_male = 1.07730065619129

Const year_coef = -1.39892516025946E-02

Const age_gender_male = -9.52692229091698E-03

Const age_gender_female = 0

Const exposure_rate_ratio_mortality As Double = 2.27

'# 1b. parameters for the hospitalisation function - modifiers (not users) could change these values

Const Intercept_h As Double = -6.06349702978915

Const age_coef_h As Double = 0.024645832496484

Const simd1_h As Double = 0.345768298654941

Const simd2_h As Double = 0.144238115229873

Const simd3_h As Double = 0

Const simd4_h As Double = -0.129426862177807

Const simd5_h As Double = -0.253367711139884

Const gender_female_h = 0

Const gender_male_h = -0.5602886786945

Const year_coef_h = 1.64061213663119E-03

Const age_gender_male_h = 1.02462431276329E-02

Const age_gender_female_h = 0

Const exposure_rate_ratio_hosp As Double = 1.52

'# 2. parameters for the rate ratio function - modifiers (not users) could change these values

'# If the intervention is harmful, ie above 1, invert.rr has to be changed to 1 (default -1)

'# for mortality

Const gain = 3.61895533940247E-03

Const threshold = -1506.15692812996

Const invert_rr = -1

'# for hospitalisation

Const gain_h = 4.08625529033277E-03

Const threshold_h = -1381.45250884331

Const invert_rr_h = -1

'

'# Function to make cumulative incidence from integrand

Function cumulativeincidenceF(integrand) As Double

cumulativeincidenceF = 1 - Exp(-integrand)

End Function

' Integration function, note that this is built in to R, and takes

' the relevant mortality function (eg mortality_rr) and time as an argument, in VBA we have "if" statements with each mortality function coded into this function

' as VBA can't take an argument saying "use this function". This bespoke function essentially replicates the funcionality of the R "integrate" function

Function IntegrateF(a As Double, b As Double, age_start As Integer, _

sex As String, simdquintile As Integer, year_start As Integer, _

mortality_function As String, total As Double, exposed As Integer, Optional N As Integer = 10) As Double

'This function calculates the area under the curve y(x) from

'x=a to x=b using Simpson's rule with n intervals

'Note: n must be even

'

'Local variables

Dim h As Double, sum As Double, term As Double

Dim x As Double

Dim i As Integer

Dim Simpson As Double

Dim prevalence_exposed As Double

' calculate prevalence exposed

prevalence_exposed = exposed / total

'Do error checking

If N = 0 Or N Mod 2 = 1 Then

Simpson = 0#

MsgBox "Sorry # of intervals has to be > 0 and even"

Exit Function

End If

h = (b - a) / N

x = a

sum = 0#

For i = 1 To N Step 2

If mortality_function = "mortality_rate" Then term = _

(mortality_rate(x, age_start, sex, simdquintile, year_start) + _

4 * mortality_rate(x + h, age_start, sex, simdquintile, year_start) + _

mortality_rate(x + 2 * h, age_start, sex, simdquintile, year_start)) _

* exposure_rate_ratio_mortality / (prevalence_exposed * exposure_rate_ratio_mortality + 1 - prevalence_exposed)

If mortality_function = "mortality_rate_rr" Then term = _

(mortality_rate_rr(x, age_start, sex, simdquintile, year_start) + _

4 * mortality_rate_rr(x + h, age_start, sex, simdquintile, year_start) + _

mortality_rate_rr(x + 2 * h, age_start, sex, simdquintile, year_start)) _

* exposure_rate_ratio_mortality / (prevalence_exposed * exposure_rate_ratio_mortality + 1 - prevalence_exposed)

If mortality_function = "mortality_rate_unex" Then term = (1 / exposure_rate_ratio_mortality) * _

(mortality_rate(x, age_start, sex, simdquintile, year_start) + _

4 * mortality_rate(x + h, age_start, sex, simdquintile, year_start) + _

mortality_rate(x + 2 * h, age_start, sex, simdquintile, year_start)) _

* exposure_rate_ratio_mortality / (prevalence_exposed * exposure_rate_ratio_mortality + 1 - prevalence_exposed)

sum = sum + term

x = x + 2 * h

Next i

Simpson = sum * h / 3

IntegrateF = Simpson

End Function

'# Mortality rate, hospitalisation rate and rate ratio functions

'# these will be integrated with respect to follow-up time note that

'# the coefficients and age at baseline are constants from the point of view of the integration

Function rr(time As Double)

rr = 1 / (1 + Exp(-gain * (time - threshold)))

rr = rr ^ (-invert_rr)

End Function

Function rr_h(time As Double)

rr_h = 1 / (1 + Exp(-gain_h * (time - threshold_h)))

rr_h = rr_h ^ (-invert_rr_h)

End Function

Function mortality_rate(time As Double, age_start As Integer, sex As String, simdquintile As Integer, year_start As Integer) As Double

Dim gender As Double

Dim gender_inter As Double

Dim simd As Double

If sex = "female" Then gender = gender_female

If sex = "female" Then gender_inter = age_gender_female

If sex = "male" Then gender = gender_male

If sex = "male" Then gender_inter = age_gender_male

If simdquintile = 1 Then simd = simd1

If simdquintile = 2 Then simd = simd2

If simdquintile = 3 Then simd = simd3

If simdquintile = 4 Then simd = simd4

If simdquintile = 5 Then simd = simd5

mortality_rate = Exp(Intercept + simd + gender + age_coef * (age_start + time) + year_coef * (year_start + time) + gender_inter * (age_start + time))

End Function

' Hospitalisation rate function, takes same form and has same user arguments as mortality function, different coefficient values

Function hosp_rate(time As Double, age_start As Integer, sex As String, simdquintile As Integer, year_start As Integer) As Double

Dim gender As Double

Dim gender_inter As Double

Dim simd As Double

If sex = "female" Then gender = gender_female_h

If sex = "female" Then gender_inter = age_gender_female_h

If sex = "male" Then gender = gender_male_h

If sex = "male" Then gender_inter = age_gender_male_h

If simdquintile = 1 Then simd = simd1_h

If simdquintile = 2 Then simd = simd2_h

If simdquintile = 3 Then simd = simd3_h

If simdquintile = 4 Then simd = simd4_h

If simdquintile = 5 Then simd = simd5_h

hosp_rate = Exp(Intercept_h + simd + gender + age_coef_h * (age_start + time) + year_coef_h * (year_start + time) + gender_inter * (age_start + time))

End Function

Function mortality_rate_rr(time As Double, age_start As Integer, sex As String, simdquintile As Integer, year_start As Integer) As Double

mortality_rate_rr = mortality_rate(time, age_start, sex, simdquintile, year_start) * rr(time)

End Function

Function hosp_rate_rr(time As Double, age_start As Integer, sex As String, simdquintile As Integer, year_start As Integer) As Double

hosp_rate_rr = hosp_rate(time, age_start, sex, simdquintile, year_start) * rr_h(time)

End Function

'# This function calcualtes the cumulative incidence for each group

Function new_ci(time As Double, age_start As Integer, sex As String, simdquintile As Integer, year_start As Integer, _

treated_each_year As Integer, n_years_treatment As Integer, total As Double, exposed As Integer, Optional CI_output As String = "Total CI") As Variant

Dim integrand_unexposed

Dim integrand_untreated

Dim integrand_tx_base

Dim integrand_treated

Dim cumulative_incidence_unexposed

Dim cumulative_incidence_untreated

Dim cumulative_incidence_tx_base

Dim cumulative_incidence_treated

Dim i As Integer

Dim j As Double

Dim ci As Double

Dim tmparray(1 To 3) As Variant

'# First, the cumulative incidence in people who are not exposed to the risk factor

'# produces a 1 column wide table with the number of new cases, number of rows correspond to length of follow-up plus 1

'# Input is the mortality rate in the unexposed (mortality rate overall * rate ratio at time zero)

'# loops through integrating the mortality rate function and transforming (CI = 1- exp(integrand) ) in order

'# to calculate the proportion of new cases at each point in FU

'# Multiplies this proportion by the number unexposed at baseline to get the cumulative incidence

integrand_unexposed = IntegrateF(1, time, age_start, sex, simdquintile, year_start, "mortality_rate_unex", total, exposed)

cumulative_incidence_unexposed = cumulativeincidenceF(integrand_unexposed) * (total - exposed)

'# Secondly, the cumulative incidence in the untreated. Similar to CI in the unexposed,

'# this calculates the CI at each time point using the mortality rate and the number exposed but untreated

integrand_untreated = IntegrateF(1, time, age_start, sex, simdquintile, year_start, "mortality_rate", total, exposed)

cumulative_incidence_untreated = cumulativeincidenceF(integrand_untreated) * (exposed - treated_each_year * n_years_treatment)

'# Thirdly, the cumulative incidence IF THOSE TREATED UNDER THE SCENARIO HAD NOT BEEN TREATED, ie THE BASE CASE FOR THE SCENARIO

integrand_tx_base = integrand_untreated

cumulative_incidence_tx_base = cumulativeincidenceF(integrand_tx_base) * treated_each_year * n_years_treatment

'# Fourthly, and much more complicatedly, the CI in the treated people.

'# As with loops 1, 2 and 3 this code calculates the CI at each time point corresponding to each year of follow-up

'# However, it does so for n groups, where n is the number of years at which treatments were applied.

'# The number of columns correspond to the number of groups

'# For example, if there were 3 years of treatment, there are 3 groups, and the output will be a CI table with 3 columns

'# For each group, this code integrates the function for the mortality rate for the exposed group until the treatment starts

'# after the treatment starts it then integrates the function for the mortality rate for the exposed group x the rate ratio for the treatment

'# taking into account how the effect of the treatment attenuates (or increases) over time

'# thus it is produces a matrix rather than a single column

For i = 1 To n_years_treatment

If i > time Then j = time

If i <= time Then j = i

integrand_untreated = IntegrateF(1, j, age_start, sex, simdquintile, year_start, "mortality_rate", total, exposed)

integrand_treated = IntegrateF(j, time, age_start, sex, simdquintile, year_start, "mortality_rate_rr", total, exposed)

ci = cumulativeincidenceF(integrand_untreated + integrand_treated) * treated_each_year

cumulative_incidence_treated = cumulative_incidence_treated + ci

Next i

' The R code produces a matrix of cumulative incidences by group, then this is later summarised

' Within the VBA code the summary is all done within this new_CI bespoke function

' Users can choose what output they want, the total cumulative incidence, the treated group CI, the untreated group, the unexposed group

' and an array with all three groups combined - this latter is used for calculating the number of hospitalisations

If CI_output = "Total CI" Then new_ci = cumulative_incidence_unexposed + cumulative_incidence_untreated + cumulative_incidence_treated

If CI_output = "unexposed" Then new_ci = cumulative_incidence_unexposed

If CI_output = "untreated" Then new_ci = cumulative_incidence_untreated

If CI_output = "treated" Then new_ci = cumulative_incidence_treated

tmparray(1) = cumulative_incidence_unexposed

tmparray(2) = cumulative_incidence_untreated

tmparray(3) = cumulative_incidence_treated

If CI_output = "Each CI" Then new_ci = tmparray

End Function

'Function to calculate Yll - Loops through the cumulative incidence calculating the years of life lost as (100 - age at death) x number dying each year.

Function new_yll(ylltime As Double, age_start As Integer, sex As String, simdquintile As Integer, year_start As Integer, _

treated_each_year As Integer, n_years_treatment As Integer, total As Double, exposed As Integer) As Double

Dim i As Double

Dim ci As Double

Dim ci_last As Double

Dim yll As Double

ylltime = ylltime - 1

ci_last = 0

For i = 1 To ylltime

ci = new_ci(i + 1, age_start, sex, simdquintile, year_start, treated_each_year, n_years_treatment, total, exposed)

yll = (ci - ci_last) * (100 - age_start - i)

new_yll = new_yll + yll

ci_last = ci

Next i

End Function

' Function to calculate hospitalisations - As for years of life lost this loops through the cumulative incidence function.

' This does so by calculating the person time each year of follow-up and multiplying this by the appropraite hosptialisation rate (unexposed, exposed or treated)

Function hosp_count(hosptime As Double, age_start As Integer, sex As String, simdquintile As Integer, year_start As Integer, _

treated_each_year As Integer, n_years_treatment As Integer, total As Double, exposed As Integer) As Double

Dim i As Double

Dim ci_each() As Variant

Dim ci_untreated As Double

Dim ci_untreated_last As Double

Dim hosp_untreated As Double

Dim ci_treated As Double

Dim ci_treated_last As Double

Dim hosp_treated As Double

Dim ci_unexposed As Double

Dim ci_unexposed_last As Double

Dim hosp_unexposed As Double

Dim unexposed_N As Double

Dim treated_N As Double

Dim untreated_N As Double

Dim prevalence_exposed_hosp As Double

' define prevalence exposed (change variable name so no class with IntegrateF

prevalence_exposed_hosp = exposed / total

hosptime = hosptime - 1

ci_unexposed_last = 0

ci_untreated_last = 0

ci_treated_last = 0

unexposed_N = total - exposed

treated_N = treated_each_year * n_years_treatment

untreated_N = total - unexposed_N - treated_N

For i = 1 To hosptime

ci_each = new_ci(i, age_start, sex, simdquintile, year_start, treated_each_year, n_years_treatment, total, exposed, "Each CI")

ci_unexposed = ci_each(1)

ci_untreated = ci_each(2)

ci_treated = ci_each(3)

hosp_untreated = (untreated_N - ci_untreated) * hosp_rate(i, age_start, sex, simdquintile, year_start) _

* exposure_rate_ratio_hosp / (prevalence_exposed_hosp * exposure_rate_ratio_hosp + 1 - prevalence_exposed_hosp)

hosp_treated = (treated_N - ci_treated) * hosp_rate_rr(i, age_start, sex, simdquintile, year_start) _

* exposure_rate_ratio_hosp / (prevalence_exposed_hosp * exposure_rate_ratio_hosp + 1 - prevalence_exposed_hosp)

hosp_unexposed = (unexposed_N - ci_unexposed) * (1 / exposure_rate_ratio_hosp) * hosp_rate(i, age_start, sex, simdquintile, year_start) _

* exposure_rate_ratio_hosp / (prevalence_exposed_hosp * exposure_rate_ratio_hosp + 1 - prevalence_exposed_hosp)

hosp_count = hosp_count + hosp_unexposed + hosp_untreated + hosp_treated

ci_unexposed_last = ci_unexposed

ci_untreated_last = ci_untreated

ci_treated_last = ci_treated

Next i

End Function
