# Supplementary material for: Informing Investment to Reduce Inequalities: A Modelling Approach
Source: PLoS One. 2016 Aug 3;11(8):e0159256. doi: 10.1371/journal.pone.0159256 (PMC4972318; doi:10.1371/journal.pone.0159256)
Supplement: S2 Appendix — (DOCX) [file pone.0159256.s002.docx]

Appendix A

25^th^ March 2016

To whom it may concern

**Re: Permission from the Scottish Public Health Observatory to republish information**

I am writing to confirm that material from the ScotPHO website relating to the ‘Informing Investment to reduce Inequalities (III)’ project can be reproduced by the Plos One under your CC BY 4.0 license.

Yours sincerely


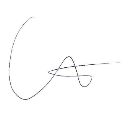


**Dr Gerry McCartney**

Public Health Consultant

Public Health Science Directorate

NHS Health Scotland

Meridian Court

5 Cadogan Street

Glasgow G2 6QE

🕿phone 0141 414 2750 🖃 email: [gmccartney@nhs.net](mailto:gmccartney@nhs.net)  website: [www.healthscotland.com](http://www.healthscotland.com/)

Our team is part of the ScotPHO collaboration, providing public health information for health improvement: [www.scotpho.org.uk](http://www.scotpho.org.uk/)
