# Supplementary material for: Informing Investment to Reduce Inequalities: A Modelling Approach
Source: PLoS One. 2016 Aug 3;11(8):e0159256. doi: 10.1371/journal.pone.0159256 (PMC4972318; doi:10.1371/journal.pone.0159256)
Supplement: S1 Table — (DOCX) [file pone.0159256.s003.docx]

Table A: Exposure risk ratios and proportion of Population at Risk (PAR) eligible for treatment for each model

|  | **Exposure risk ratio** | | **Percentage of the PAR eligible for the intervention** | |
| --- | --- | --- | --- | --- |
|  | **Mortality** | **Hospitalisation** | **%** | **Rationale** |
| **Tobacco tax** | 2.27^[[1]](#endnote-1),^^[[2]](#endnote-2)^ | 1.52^[[3]](#endnote-3)^ | 80% | Assume 20% of the PAR (i.e. aged 16 years or more) unaffected due to increases in illegal sources of tobacco, and that this applies equally across SIMD quintiles.^[[4]](#endnote-4)^ |
| **Smoking cessation** | 2.27^i,ii^ | 1.52^iii^ | 74% | 74% of the PAR eligible for the intervention (i.e. aged >16 years) using data on quit motivation.^[[5]](#endnote-5)^ |
| **ABIs** | 1.25^[[6]](#endnote-6)^ | 1.10.^[[7]](#endnote-7)^ | 39% | 39% of hazardous/harmful drinkers aged >16 years want to reduce alcohol consumption.^v^ |
| **Counterweight** | 1.39^[[8]](#endnote-8)^ | 1.18^iii^ | 71% | We used data on ‘motivation to lose weight’ among the population who were obese aged >16 years.^v^ |
| **Employment** | 1.63^[[9]](#endnote-9)^ | 1.02^[[10]](#endnote-10)^ | 34% | Assume all unemployed (i.e. aged 15–69 years) and 30% of those aged 15–24 years who are economically inactive and not in full-time education are eligible. |
| **Active travel** | 1.30^[[11]](#endnote-11)^ | No evidence identified for modelling | 33% | Intervention only relevant to those aged 16–64 years who commute <3 miles by car or van based on data.^[[12]](#endnote-12)^ |

1. Iversen L, Fielding S, Hannaford PC. Smoking in young women in Scotland and future burden of hospital admission and death: a nested cohort study. British Journal of General Practice. 2013; 63(613): e523–33. [↑](#endnote-ref-1)
2. Doll R, Peto R, Boreham J, Sutherland I. Mortality in relation to smoking: 50 years’ observations on male British doctors. British Medical Journal. 2004; 328: 1519–33. [↑](#endnote-ref-2)
3. Lawder R, Elders A, Clark D. Using the Linked Scottish Health Survey to Predict Hospitalisation & Death. Edinburgh, ScotPHO, 2007. [↑](#endnote-ref-3)
4. Guindon GE, Driezen P, Chaloupka FJ, Fong GT. Cigarette tax avoidance and evasion: findings from the International Tobacco Control Policy Evaluation Project. Tobacco Control. 2013; 0: 1–10, doi:10.1136/tobaccocontrol-2013-051074 [↑](#endnote-ref-4)
5. Rutherford L, Reid S. Knowledge, attitudes and motivations to health, 2008–11. Edinburgh, NHS Health Scotland, 2013. [↑](#endnote-ref-5)
6. Stringhini S, Dugravot A, Shipley M, Goldberg M, Zins M, Kivimäki M, et al. Health Behaviours, Socio-economic Status, and Mortality: Further Analyses of the British Whitehall II and the French GAZEL Prospective Cohorts. PLoS Medical. 2011; 8(2): e1000419. [↑](#endnote-ref-6)
7. Hart CL & Smith GD. Alcohol consumption and mortality and hospital admissions in men from the Midspan collaborative cohort study. Addiction. 2008; 103(12): 1979–86. [↑](#endnote-ref-7)
8. Prospective Studies Collaboration. Body-mass index and cause-specific mortality in 900 000 adults: collaborative analyses of 57 prospective studies. Lancet. 2009; 373(9669): 1083–96. [↑](#endnote-ref-8)
9. Roelfs DJ, Shor E, Davidson KW, Schwartz JE. Losing Life and Livelihood: A Systematic Review and Meta- Analysis of Unemployment and All-Cause Mortality. Social Science and Medicine. 2011; 72(6): 840–854. [↑](#endnote-ref-9)
10. Browning M, Heinesen, E. Effect of job loss due to plant closure on mortality and hospitalization. Journal of Health Economics. 2012; 31: 599–616. [↑](#endnote-ref-10)
11. Department of Health, Physical Activity, Health Improvement and Protection. Start Active, Stay Active: A report on physical activity from the four home countries’ Chief Medical Officers. London: Department of Health, 2011. [↑](#endnote-ref-11)
12. Office for National Statistics. 2001 Census. Available: <http://www.ons.gov.uk/ons/guide-method/census/census-2001/index.html> [↑](#endnote-ref-12)
