# Supplementary material for: Informing Investment to Reduce Inequalities: A Modelling Approach
Source: PLoS One. 2016 Aug 3;11(8):e0159256. doi: 10.1371/journal.pone.0159256 (PMC4972318; doi:10.1371/journal.pone.0159256)
Supplement: S2 Table — (DOCX) [file pone.0159256.s004.docx]

Table A: Summary of evidence and assumptions used for intervention impact

| **Intervention** | **Assumed intervention effectiveness** | **Assumed change in intervention risk ratio over time** | **Other assumptions** |
| --- | --- | --- | --- |
| **Income** | Varies by income intervention; dependent on the percentage change in mean household income experienced by each SIMD quintile. | Intervention risk ratio is assumed constant over time. | Standardised mortality and hospitalisation rates by SIMD income domain quintile can be applied to income quintile and overall SIMD quintile; mean income by SIMD quintile matches mean income by income quintile; the income/health relationship is not confounded. |
| **Tobacco tax** | The reduction in smoking prevalence falls to 1.4% after 2 years before stabilising (based on a price elasticity of demand for tobacco in high income countries (including the UK) of between -0.2 and -0.6^[[1]](#endnote-1)^ and a relapse rate derived from studies of nicotine replacement^[[2]](#endnote-2)^). | Smoking cessation risk ratios increase from 0.99 to 0.72 (mortality) and from 0.99 to 0.83 (hospitalisation) and do not vary across population strata.^[[3]](#endnote-3)^,^[[4]](#endnote-4),^^[[5]](#endnote-5)^ | 11.5% of the PAR (>16 years) will stop smoking without the intervention in a linear fashion over 20 years.^[[6]](#endnote-6)^ |
| **Smoking cessation** | 7% of the treated group abstinent after one year, falling to 4.9% after two years then stabilising (based on five-year averages from the Scottish smoking cessation database). | As for tobacco tax. | As for tobacco tax. |
| **ABIs** | 65% of those receiving the intervention (>15 years) comply and have a successful outcome, (based on a loss-to-follow up estimate from similar interventions^[[7]](#endnote-7)^ - those lost to follow-up derive no benefit) defined as a decrease of 3.66 units of alcohol per week.^[[8]](#endnote-8)^ | Intervention risk ratios for successful interventions of 0.97 for mortality and 0.95 for hospitalisation were used,^[[9]](#endnote-9)^ returning linearly to 1.00 over seven years (and the same across all population strata).^[[10]](#endnote-10)^ | The PAR in the untreated group will remain static over the 20-year period. |
| **Counterweight** | 40% of those receiving the intervention (>15 years) comply and have a successful outcome (those lost to follow-up derive no benefit) defined as a mean loss of 3.7 kg (1.36 kg/m^2^) at 12 months.^[[11]](#endnote-11)^ | The population experiencing a successful intervention have a risk ratio of 0.91 for mortality^[[12]](#endnote-12)^ and 0.93^[[13]](#endnote-13)^ for hospitalisation, returning linearly to 1.00 over five years (and the same across all population strata). | The PAR in the untreated group will increase linearly by 14.5% over the 20-year period, based on obesity projections by the Scottish Government (2010).^[[14]](#endnote-14)^  In the absence of intervention obese individuals increase weight by 1 kg per year.^[[15]](#endnote-15)^ |
| **Employment** | 75% of the ‘treated’ group will remain in employment after one year; 67% after 20 years.^[[16]](#endnote-16)^ | The intervention risk ratio on mortality decreases (0.56 after one year, 0.90 after 20 years).^[[17]](#endnote-17)^ Similar assumptions for hospitalisations had a negligible impact. | Each year, 1.6% of the non-intervention group (16–69 years) move into employment (34% of the population at risk over 20 years) (Office for National Statistics (ONS) UK labour market flow data.^[[18]](#endnote-18)^) |
| **Active travel** | One-third of eligible commuters (aged 16–64 years, 11% of the PAR) increase PA by 120 minutes per week (iConnect study^^[[19]](#endnote-19)^^). Every additional 15 minutes PA beyond the first 15 per day reduces mortality by 4%; the effect the same for all population strata.^[[20]](#endnote-20)^ | Intervention risk ratio is constant over time. | The impact of increased injuries or health impacts of air pollution are insignificant compared to the impacts of changes in PA.^[[21]](#endnote-21),^^[[22]](#endnote-22)^ |

1. IARC Handbooks of Cancer Prevention, Tobacco Control; Vol 14: Effectiveness of Tax and price Policies for Tobacco Control. Lyon, IARC, 2011. [↑](#endnote-ref-1)
2. Etter, J-F & Satpleton JA. Nicotine replacement therapy for long-term smoking cessation: a meta-analysis. Tobacco Control. 2006; 15: 280–285. [↑](#endnote-ref-2)
3. Lawder R, Elders A, Clark D. Using the Linked Scottish Health Survey to Predict Hospitalisation & Death. Edinburgh, ScotPHO, 2007. [↑](#endnote-ref-3)
4. Thun MJ, Carter BD, Feskanich D, Freedman ND, Prentice R, Lopez AD, et al. 50-Year Trends in Smoking-Related Mortality in the United States. The New England Journal of Medicine. 2013; 368: 351–64. [↑](#endnote-ref-4)
5. Hart CL, Gruer L, Bauld L. Does Smoking Reduction in Midlife Reduce Mortality Risk? Results of 2 Long-Term Prospective Cohort Studies of Men and Women in Scotland. American Journal of Epidemiology. 2013; 178(5): 770–779. [↑](#endnote-ref-5)
6. Scottish Government. Projection of smoking population for Scotland. Edinburgh, Scottish Government, 2013. [↑](#endnote-ref-6)
7. Kaner EF, Dickinson HO, Beyer FR, Campbell F, Schlesinger C, Heather N, et al. Effectiveness of brief alcohol interventions in primary care populations (Review). Cochrane Database of Systematic Reviews, 2007. [↑](#endnote-ref-7)
8. Jonas DE, Garbutt JC, Amick HR, Brown JM, Brownley KA, Council CL, et al. Behavioral Counseling After Screening for Alcohol Misuse in Primary Care: a systematic review and meta-analysis for the U.S. Preventive Services Task Force. Annals of Internal Medicine. 2012 Nov 6; 157(9): 645–54. [↑](#endnote-ref-8)
9. Hart CL & Smith GD. Alcohol consumption and mortality and hospital admissions in men from the Midspan collaborative cohort study. Addiction. 2008; 103 (12): 1979–86. [↑](#endnote-ref-9)
10. Purshouse RC, Brennan A, Rafia R, Latimer NR, Archer RJ, Angus CR, et al. Modelling the cost-effectiveness of alcohol screening and brief interventions in primary care in England. Alcohol & Alcoholism. 2013; 48(2): 180–8. [↑](#endnote-ref-10)
11. Counterweight Project Team. The implementation of the Counterweight Programme in Scotland, UK. Family Practice. 2012; 29: i139–i144. [↑](#endnote-ref-11)
12. Prospective Studies Collaboration. Body-mass index and cause-specific mortality in 900 000 adults: collaborative analyses of 57 prospective studies. Lancet. 2009; 373(9669): 1083–96. [↑](#endnote-ref-12)
13. Korda RJ, Liu B, Clements MS, Bauman AE, Jorm LR, Bambrick HJ, et al. Prospective cohort study of body mass index and the risk of hospitalisation: findings from 246 361 participants in the 45 and Up Study. International Journal of Obesity. 2013; 37: 790–799. [↑](#endnote-ref-13)
14. Scottish Government. Preventing Overweight and Obesity in Scotland: A Route Map Towards Healthy Weight. Edinburgh: Scottish Government, 2010. [↑](#endnote-ref-14)
15. Heitmann BL & Garby L. Patterns of long-term weight changes in overweight developing Danish men and women aged between 30 and 60 years. International Journal of Obesity. 1999; 23(10):1074–1078. [↑](#endnote-ref-15)
16. Adams L, Oldfield K, Riley C, Skone James A. Destinations of Jobseeker’s Allowance, Income Support and Employment and Support Allowance Leavers 2011. Department for Work and Pensions Research Report No 791. DWP, Sheffield, 2012. [↑](#endnote-ref-16)
17. Browning M, Heinesen, E. Effect of job loss due to plant closure on mortality and hospitalization. Journal of Health Economics. 2012; 31: 599–616. [↑](#endnote-ref-17)
18. Labour Force Survey Five-Quarter Longitudinal Dataset, January 2011 - March 2012. Available: <http://discover.ukdataservice.ac.uk/catalogue/?sn=7035> [↑](#endnote-ref-18)
19. Sahlqvist S, Goodman A, Cooper AR, Ogilvie D. Change in active travel and changes in recreational and total physical activity in adults: longitudinal findings from the iConnect study. International Journal of Behavioral Nutrition and Physical Activity. 2013; 10: 28. [↑](#endnote-ref-19)
20. Wen CP, Wai JP, Tsai MK, Yang YC, Cheng TY, Lee MC, et al. Minimum amount of physical activity for reduced mortality and extended life expectancy: a prospective cohort study. Lancet. 2011; 378: 1244–53. [↑](#endnote-ref-20)
21. de Hartog JJ, Boogaard H, Nijlandet H, Hoek G. Do The Health Benefits Of Cycling Outweigh The Risks? Environmental Health Perspectives. 2010; 118(8): 1109–16. [↑](#endnote-ref-21)
22. Woodcock J, Edwards P, Tonne C, Armstong BG, Ashiru O, Banister D, et al. Public health benefits of strategies to reduce greenhouse-gas emissions: urban land transport. Lancet. 2009; 374: 1930–43. [↑](#endnote-ref-22)
