# Supplementary material for: Informing Investment to Reduce Inequalities: A Modelling Approach
Source: PLoS One. 2016 Aug 3;11(8):e0159256. doi: 10.1371/journal.pone.0159256 (PMC4972318; doi:10.1371/journal.pone.0159256)
Supplement: S3 Table — (DOCX) [file pone.0159256.s005.docx]

Table A: Level of intervention required to match absolute impact of introduction of Living Wage, up to 100% recruitment of eligible population

| **Intervention** | **Years of life saved** | **Intervention** | **Hospitalisations prevented** |
| --- | --- | --- | --- |
| Living Wage | 76,851 | Living Wage | 56,444 |
| If 147,000 people found work | 76,420 | If 100% of smokers who want to quit recruited (734,000) | 22,537 |
| If 100% of smokers who want to quit recruited (734,000) | 36,555 | If 100% of those who want to control weight recruited to counterweight (855,000) | 20,531 |
| If 100% of those who want to control weight recruited to counterweight (855,000) | 27,435 | 10% rise in JSA/IS | 16,843 |
| 10% rise in JSA/IS | 26,101 | If 100% of those eligible found work (368,000) | 9,164 |
| If 100% of non-active commuters were to switch (480,000) | 11,784 | If 100% of those who want to cut down alcohol participate in ABI (391,000) | 9,012 |
| 10% rise in tobacco price (through tax) | 11,284 | 10% rise in tobacco price (through tax) | 6,960 |
| 10% rise in Working Tax Credit | 8,114 | 10% rise in Working Tax Credit | 5,715 |
| If 100% of those who want to cut down alcohol participate in ABI (391,000) | 4,974 | 10% rise in Council tax | -11,940 |
| 10% rise in Council tax | -16,023 | 1p rise in income tax | -18,251 |
| 1p rise in income tax | -21,871 | If 100% of non-active commuters were to switch (480,000) | n/a |
