# Supplementary material for: Informing Investment to Reduce Inequalities: A Modelling Approach
Source: PLoS One. 2016 Aug 3;11(8):e0159256. doi: 10.1371/journal.pone.0159256 (PMC4972318; doi:10.1371/journal.pone.0159256)
Supplement: S4 Table — (DOCX) [file pone.0159256.s006.docx]

Table A: Level of intervention required to match impact on inequalities of 10% increase in JSA/IS, up to 100% recruitment of eligible population

| **Intervention** | **Years of life saved** | **Intervention** | **Hospitalisations prevented** |
| --- | --- | --- | --- |
| 10% rise in JSA/IS | -0.88 | 10% rise in JSA/IS | -0.66 |
| If 345,000 people found work | -0.88 | 1p rise in income tax | -0.41 |
| 1p rise in income tax | -0.49 | Living Wage | -0.36 |
| Living Wage | -0.32 | If 100% of smokers who want to quit recruited (734,000) | -0.23 |
| If 100% of smokers who want to quit recruited (734,000) | -0.26 | 10% rise in Working Tax Credit | -0.10 |
| 10% rise in Working Tax Credit | -0.12 | 10% rise in tobacco price (through tax) | -0.07 |
| 10% rise in tobacco price (through tax) | -0.08 | If 100% of those who want to control weight recruited to counterweight (855,000) | -0.03 |
| If 100% of those who want to control weight recruited to counterweight (855,000) | -0.01 | 10% rise in Council tax | 0.03 |
| 10% rise in Council tax | 0.03 | If 100% of those eligible found work (368,000) | 0.04 |
| If 100% of those who want to cut down alcohol participate in ABI (391,000) | 0.03 | If 100% of those who want to cut down alcohol participate in ABI (391,000) | 0.05 |
| If 100% of non-active commuters were to switch (480,000) | 0.13 | If 100% of non-active commuters were to switch (480,000) | n/a |
